# mRNA cancer vaccines: delivery strategies, immune modulation, and clinical translation

**DOI:** 10.3389/fphar.2026.1852041

**Published:** 2026-07-07

**Authors:** Dianzhe Tian, Xinshi Li, Zuyi Yang, Xinyu Zhao, Zixuan Hu, Haitao Zhao, Shunda Du, Shengzhi Liu, Lei Zhang, Yiyao Xu, Xin Lu

**Affiliations:** 1 Department of Liver Surgery, State Key Laboratory of Complex Severe and Rare Diseases, Peking Union Medical College Hospital, Chinese Academy of Medical Sciences and Peking Union Medical College, Beijing, China; 2 Eight-Year Medical Doctor Program, Chinese Academy of Medical Sciences and Peking Union Medical College, Beijing, China; 3 Department of Pharmacology, College of Pharmaceutical Sciences of Capital Medical University, Beijing, China; 4 Key Laboratory of Ocular Fundus Disease, Chinese Academy of Medical Sciences, Beijing, China; 5 Department of Ophthalmology, Peking Union Medical College Hospital, Beijing, China; 6 Beijing Area Major Laboratory of Peptide and Small Molecular Drugs, Engineering Research Center of Endogenous Prophylactic of the Ministry of Education of China, Beijing Laboratory of Biomedical Materials, Beijing, China

**Keywords:** cancer immunotherapy, delivery systems, lipid nanoparticles (LNP), mRNA vaccines, personalized therapy, tumor-specific antigens

## Abstract

Cancer remains a major global health challenge, and traditional treatments often involve significant toxicity and limited benefits for patients with advanced-stage disease. mRNA vaccines have recently emerged as a promising approach in cancer immunotherapy due to their flexibility in design, rapid production, and suitability for personalized treatment. This review summarizes the molecular basis, main classifications, and mechanisms of action of mRNA vaccines for cancer therapy and systematically discusses progress in strategies for delivering tumor-specific antigens, tumor-associated antigens, and immunomodulatory factors. Special attention is given to advances in delivery technologies, especially lipid nanoparticle (LNP) systems, and their potential applications across various cancers. We suggest that the effectiveness of mRNA cancer vaccines depends not only on selecting the right antigens but also on delivery methods that modulate the immune response and reshape the tumor microenvironment. Melanoma is a prime candidate for prioritizing the research and development of mRNA cancer vaccines, given the current clinical landscape. Furthermore, major challenges that continue to limit progress include insufficient stability, suboptimal delivery efficiency, uncontrolled immunogenicity, and the difficulty of overcoming tumor heterogeneity. This review aims to serve as a useful reference for further development and clinical translation of mRNA cancer vaccines.

## Graphical Abstract

**Figure:**
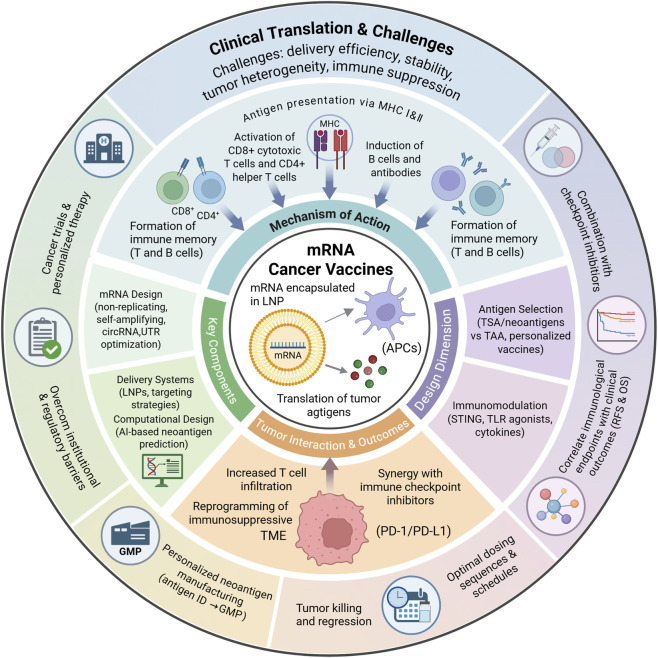

## 1 Introduction

Cancer treatment remains one of the biggest challenges in modern medicine. While traditional therapies have provided important clinical benefits, they are often limited by only moderate effectiveness, high chances of cancer returning, and notable side effects. Immunotherapy has emerged as a breakthrough approach that utilizes the host immune system to identify and destroy tumor cells, thereby supplementing traditional cancer treatments (Zafar et al., 2024). A key component of immunotherapy is innate immune activation, which bridges innate and adaptive immunity (Jiang and Lin, 2025). Recent progress in translational oncology has further highlighted the clinical importance of integrating emerging therapeutic advances into broader cancer treatment strategies (Zaidi et al., 2025).

Nevertheless, the efficacy of single-agent immunotherapies, particularly immune checkpoint inhibitors (ICIs), is often constrained by the immunosuppressive tumor microenvironment (TME) and by insufficient T-cell infiltration (Jiang and Lin, 2025; Du et al., 2026). Consequently, activating innate immunity to support and amplify adaptive immune responses has become a critical strategy for overcoming resistance to immunotherapy (Jiang and Lin, 2025). Innate immune modulators (IIMs), including agonists targeting cGAS-STING, Toll-like receptor (TLR) pathways, and RIG-I-like receptor pathways, can robustly initiate immune responses and reshape the TME, thereby offering new avenues for cancer immunotherapy (Jiang and Lin, 2025; Tabar et al., 2024). mRNA vaccines have recently gained considerable attention as a novel immunotherapeutic modality due to their flexible design, rapid production, and high potential for personalization (Li et al., 2023; Duan et al., 2022). Their mechanism of action involves delivering mRNA that encodes tumor antigens or immunomodulatory molecules into host cells, where intracellular translation produces target proteins that trigger antigen-specific humoral and cellular immune responses. To date, mRNA vaccines have entered clinical trial stages (Li et al., 2023; Vishweshwaraiah and Dokholyan, 2022; Chavda et al., 2022; Song et al., 2024; Tang Y. et al., 2025). Notably, accumulating evidence indicates that the therapeutic effectiveness of mRNA vaccines is not solely determined by the choice of antigen or by the efficiency of delivery. More focus has been placed on the role of innate immune activation caused by the delivery materials themselves, which can have a dual impact on antigen expression and T-cell priming. Excessive innate immune activation may paradoxically hinder mRNA translation and compromise the quality of the immune response. Therefore, future mRNA vaccine delivery systems should shift from simply maximizing adjuvant effects to a balanced approach that balances immune activation and antigen expression. This idea is increasingly seen as a crucial direction for advancing mRNA-based cancer vaccines.

## 2 Molecular design and functional basis of mRNA vaccines

**FIGURE 1:**
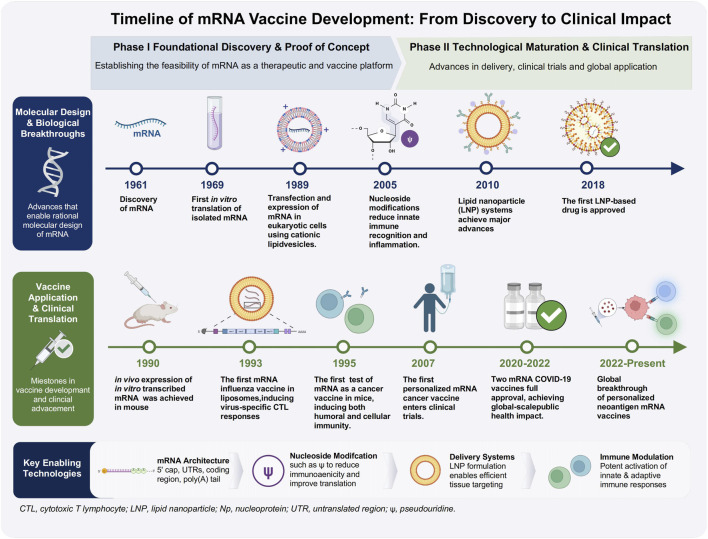
Developmental timeline and key enabling technologies of mRNA vaccines. The figure summarizes major milestones in the development of mRNA vaccines, including foundational discoveries, advances in molecular design and delivery technologies, and key steps in preclinical and clinical translation. Major enabling factors, such as mRNA architecture, nucleoside modification, LNP-based delivery, and immune modulation, are also highlighted.

The molecular design of mRNA vaccines is crucial for their effectiveness, safety, and clinical application. The timeline and key technologies for mRNA vaccine development are shown in Figure 1. Non-replicating and self-replicating mRNA are the two main types, forming the basis for mRNA cancer vaccines. Currently, non-replicating mRNA vaccines are the most widely used platform in clinical settings; they encode only the target antigen protein. Once inside host cells, these vaccines harness the cellular translational machinery to produce antigen proteins, which are then degraded without self-replication. Studies have demonstrated that lipid nanoparticle (LNP)-delivered non-replicating mRNA vaccines can effectively activate antigen-specific CD8^+^ T cells, induce memory T-cell responses, and successfully control or even eliminate established tumors in mice models (da Silva et al., 2023). Moreover, optimization of mRNA constructs can markedly enhance translational efficiency and molecular stability, thereby improving immunogenicity (Toniolo et al., 2024). Despite their proven success in inducing protective immune responses, non-replicating mRNA vaccines generally exhibit relatively short durations of antigen expression, which may necessitate higher doses or repeated administrations to achieve sustained immune protection (Lu H. H. et al., 2025; Tang L. et al., 2025). Self-replicating mRNA vaccines, also referred to as self-amplifying RNA (saRNA) vaccines, are inspired by the genomic architecture of positive-sense RNA viruses (Pourseif et al., 2022). Unlike non-replicating mRNA vaccines that encode only the antigen, saRNA constructs encode both the target antigen and viral non-structural proteins, such as RNA-dependent RNA polymerases (Vallet and Vignuzzi, 2025). Once delivered into the cytoplasm, these replicase proteins enable extensive amplification of progeny RNA using the vaccine RNA as a template, resulting in exponential intracellular expansion of the antigen-encoding sequence (Lundstrom, 2021). This self-amplification mechanism allows comparable or even higher levels of antigen expression to be achieved at substantially lower doses than those required for non-replicating mRNA vaccines (Vallet and Vignuzzi, 2025). In addition, prolonged *in vivo* antigen expression can be achieved, mimicking persistent infection and potentially eliciting more robust and durable humoral and cellular immune responses (Tang L. et al., 2025; Maruggi et al., 2022). In cancer vaccine applications, a single low-dose administration of a saRNA-LNP vaccine encoding the HPV-16 E7 oncoprotein was shown to effectively activate E7-specific CD8^+^ T cells in murine models, eliminate subcutaneous tumors at various stages of growth, and induce memory T-cell responses that prevented tumor recurrence. The therapeutic efficacy was comparable to that of optimized non-replicating mRNA vaccines (da Silva et al., 2023). However, the longer sequences and increased structural complexity of saRNA vaccines pose challenges for large-scale manufacturing, delivery system encapsulation, and molecular stability (Vallet and Vignuzzi, 2025; Yıldız et al., 2024). Furthermore, the generation of double-stranded RNA intermediates during replication may activate innate immune sensors such as TLR3, MDA5, and PKR (Pepini et al., 2017), necessitating careful regulation to balance beneficial adjuvant effects against impaired antigen translation and excessive inflammatory toxicity (Maruggi et al., 2022). Beyond linear mRNA platforms, circular RNA (circRNA) has recently emerged as an additional strategy for RNA vaccine development. Its covalently closed structure confers resistance to exonuclease-mediated degradation and may support more prolonged protein expression without requiring intracellular self-amplification (Wesselhoeft et al., 2019). In contrast to saRNA, the persistence of circRNA is mainly derived from improved molecular stability rather than replicase-driven RNA amplification, and its innate immune properties appear to depend strongly on RNA production method and purification quality, including the presence of contaminating linear RNA species or double-stranded by-products (Liu et al., 2022). Taken together, conventional non-replicating mRNA, saRNA, and circRNA present distinct trade-offs in expression kinetics, duration of antigen production, and innate immune activation. Conventional non-replicating mRNA generally enables rapid but relatively transient antigen expression because it does not undergo intracellular amplification, whereas saRNA supports more sustained antigen production at lower input doses through replicase-driven RNA amplification but also induces stronger innate sensing. circRNA, by contrast, offers enhanced structural stability and expression persistence, while its immunological behavior appears to be more dependent on RNA purity and production strategy (Wesselhoeft et al., 2019; Pardi et al., 2018; Lundstrom, 2020)

The selection of tumor-specific antigens (TSAs) and tumor-associated antigens (TAAs) is critical for mRNA vaccine design. TSAs are considered ideal targets for personalized mRNA vaccines (Huang et al., 2024). Neoantigens derived from somatic mutations are promising targets for personalized mRNA vaccines. Their identification typically depends on next-generation sequencing combined with bioinformatics algorithms that predict mutant epitopes capable of binding human leukocyte antigen (HLA) molecules and eliciting T-cell responses (Floudas et al., 2025). More broadly, emerging computational approaches, including machine learning-based antigen prioritization, RNA structure-informed sequence optimization, and integrated *in silico* design workflows, are increasingly being used to support the rational design and iterative refinement of mRNA vaccine candidates, thereby strengthening the translational potential of this platform (Zhang H. et al., 2026). However, accurately predicting immunogenic neoantigens remains challenging, and this prediction is further limited by HLA restriction. In contrast, TAAs enable the development of off-the-shelf vaccines for broader patient populations but are limited by immune tolerance, resulting in reduced immunogenicity and potential off-tumor toxicity (Bhattacharya et al., 2026). Beyond encoding tumor antigens, mRNA platforms can also deliver immunomodulatory factors and chimeric antigen receptors (CARs). These strategies aim to reshape the tumor microenvironment (TME) and enhance antitumor immunity. For example, combining antigen-encoding mRNA with STING agonists has been shown to improve vaccine efficacy (Zhang Z. et al., 2026). mRNA-based CAR strategies have also attracted increasing attention. mRNA enables transient CAR expression in T cells for targeted cytotoxicity, avoiding genomic integration and improving safety. Transient expression may also reduce toxicity and T-cell exhaustion. However, the durability of transient CAR expression in solid tumors remains uncertain.

Recent progress in mRNA vaccine engineering for cancer immunotherapy suggests several key directions that deserve particular attention. The detailed optimization strategies for mRNA vaccine design are summarized in Table 1. At the same time, the balance between stability and immunogenicity has emerged as a central issue in mRNA design. This is particularly evident in the debate over nucleoside modification. Foundational studies demonstrated that incorporation of modified nucleosides such as pseudouridine into in vitro-transcribed mRNA can suppress Toll-like receptor-mediated innate immune recognition while improving translational capacity and biological stability, thereby laying the conceptual foundation for nucleoside-modified mRNA platforms, including the later use of N1-methylpseudouridine (m1Ψ) in prophylactic infectious disease vaccines (Karikó et al., 2005; Karikó et al., 2008). However, this design principle does not translate directly to therapeutic cancer vaccines. In oncology settings, complete suppression of innate immune sensing may not always be desirable, because a controlled degree of innate activation can provide beneficial adjuvant effects. Consistent with this rationale, BioNTech’s cancer vaccine platforms, including FixVac (BNT111) and autogene cevumeran (BNT122), have employed uridine-unmodified mRNA formats to preserve innate immune stimulation, promote dendritic cell activation and type I interferon-associated signaling, and thereby support more effective T-cell priming (Beck et al., 2021; Kranz et al., 2016). By contrast, prophylactic infectious disease vaccines generally prioritize maximal antigen expression with reduced excessive innate sensing and reactogenicity, favoring the use of m1Ψ-modified mRNA. Thus, the choice between nucleoside-modified and uridine-unmodified mRNA should be viewed as a context-dependent design decision rather than a universal optimization strategy, and this distinction is especially important in the development of mRNA cancer vaccines. In certain settings, controlled innate immune activation can provide beneficial adjuvant effects, supporting dendritic cell priming and antitumor T-cell responses. Achieving a context-dependent equilibrium between antigen expression and immune stimulation will remain a critical challenge for future development. Even with optimized antigen selection and delivery, therapeutic efficacy may be limited by tumor evolution. Intratumoral heterogeneity and immunoediting can drive antigen loss and tumor escape. Therefore, the clinical success of mRNA cancer vaccines will depend on advances in antigen prediction, patient selection, and response durability.

**TABLE 1:** Optimization strategies for mRNA vaccine design.

Optimization category	Core technical strategies and approaches	Expected outcomes and functional advantages
1. mRNA molecular engineering	1. 5′ cap optimization: Adoption of Cap1/Cap2 structures and anti-reverse cap analogs (ARCA) 2. UTR engineering: Systematic optimization of length, secondary structure, and regulatory motifs 3. Poly(A) tail design: Precise control of tail length or segmented architectures 4. Nucleoside modification: Incorporation of modified nucleosides 5. Codon and structure tuning: Adjustment of GC content, elimination of rare codons, and reduction of TLR-binding motifs or ribosomal stalling sites	1. Synergistically enhances translation initiation efficiency and mRNA stability 2. Prolongs intracellular half-life by resisting exonuclease-mediated degradation and antiviral clearance 3. Improves overall protein expression levels, kinetics, fidelity, and immunogenicity in both non-replicating and self-replicating platforms
2. RNA platform design	1. Non-replicating mRNA: Encoding solely the target antigen sequence, relying on host translational machinery without self-replication 2. Self-amplifying RNA (saRNA): Incorporation of viral replicon elements for intracellular amplification 3. Circular RNA (circRNA): Generation of covalently closed RNA *via* ribozyme- or intron-mediated circularization 4. Trans-amplifying systems: Separation of replicase and antigen-encoding constructs	1. Non-replicating: Simple structure, favorable safety profile, and rapid development; effective for infectious diseases and cancer, though with shorter antigen expression duration 2. saRNA: Enables high-level antigen expression at lower doses with prolonged persistence and intrinsic adjuvant effects 3. circRNA: Provides superior structural stability and sustained antigen exposure4. Achieves dose-sparing while balancing expression magnitude and controlled immune stimulation
3. Antigen design	1. Tumor-specific antigens (TSAs/Neoantigens): Personalized identification *via* tumor sequencing, immunogenomics, and bioinformatics prediction (accounting for proteasomal processing, TAP transport, and MHC-I loading) 2. Tumor-associated antigens (TAAs): Shared antigens enabling off-the-shelf vaccines 3. Multi-epitope design: Concatenation of CD4^+^/CD8^+^ T-cell epitopes using flexible linkers4. Chimeric constructs: Fusion with trafficking signals to enhance presentation	1. TSAs: High tumor specificity, low off-target risk, and reduced central immune tolerance; ideal for personalized vaccines but limited by HLA restriction and manufacturing timelines 2. TAAs: Broader patient applicability but subject to immune tolerance and potential off-tumor toxicity 3. Induces broad polyclonal T-cell responses to mitigate immune escape4. Improves antigen processing efficiency and MHC presentation
4. Immunomodulatory strategies	1. Encoded immunomodulators: Co-expression of cytokines, co-stimulatory molecules, or STING pathway activators 2. Combination approaches: Co-delivery with STING/TLR agonistsor checkpoint inhibitors 3. CAR mRNA strategies: Transient *in vivo*/*ex vivo* expression of chimeric antigen receptors targeting tumor antigens	1. Reshapes the immunosuppressive tumor microenvironment *via* type I IFN production, dendritic cell maturation, and enhanced CD8^+^ T-cell cross-priming 2. Breaks immune tolerance and promotes antigen cross-presentation 3. Delivers potent antitumor cytotoxicity while avoiding genomic integration risks, reducing cytokine release syndrome and T-cell exhaustion
5. Enabling computational technologies	1. Deep learning models: Prediction of MHC binding affinity, immunogenicity, and epitope processing steps 2. Structural simulation: Algorithms for mRNA secondary structure prediction 3. Automated design pipelines: End-to-end computational workflows integrating sequencing, neoantigen prioritization, and optimization	1. Accelerates iterative design and optimization of personalized vaccines within therapeutic timelines 2. Reduces false-positive predictions in neoantigen identification 3. Enhances mRNA stability and translational performance through *in silico* approaches

ARCA, anti-reverse cap analog; CD4^+^, cluster of differentiation 4 positive; CD8^+^, cluster of differentiation 8 positive; circRNA, circular RNA; CRS, cytokine release syndrome; GPC3, glypican-3; HLA, human leukocyte antigen; IFN, interferon; LAMP, lysosome-associated membrane protein; MHC-I, major histocompatibility complex class I; RdRP, RNA-dependent RNA, polymerase; saRNA, self-amplifying RNA; STING, stimulator of interferon genes; TAA, tumor-associated antigen; TLR, Toll-like receptor; TSA, tumor-specific antigen; TME, tumor microenvironment.

## 3 Mechanisms of mRNA vaccine-mediated anti-tumor immune activation

### 3.1 Endogenous antigen expression and dual MHC presentation

The core mechanism of mRNA vaccines in cancer therapy is the efficient induction of antigen expression and the activation of specific cellular immune responses. Upon *in vivo* delivery, mRNA encoding tumor antigens must first enter the cytoplasm of antigen-presenting cells (APCs), a process typically reliant on efficient delivery systems (Yao et al., 2024). Examples include mRNA vaccines targeting human papillomavirus (HPV)-associated cancers and liposome-encapsulated LMP2 mRNA vaccines for nasopharyngeal carcinoma. This mode of antigen synthesis is critical for subsequent immune activation. Studies indicate that CD4^+^ T cell responses induced by mRNA-LNP vaccines depend on endogenously produced antigens within APCs rather than exogenous uptake, thereby optimizing MHC class II-restricted processing and presentation (Rood et al., 2026). The expression of synthesized antigenic proteins in APCs facilitates the activation of both innate and adaptive immunity (Miao et al., 2021).

CD8^+^ T cells exert cytotoxic effects by recognizing and killing tumor cells that express the corresponding antigens (Li et al., 2024). In tumor models, mRNA vaccines targeting HPV E6/E7 induced robust antigen-specific CD8^+^ T cell responses; these cells were detected in the spleen, peripheral blood, and tumor tissues and were pivotal for achieving complete tumor regression (Li et al., 2024). Antigen expression also promotes adaptive immune activation, including CD4^+^ T cell engagement (Miao et al., 2021). To further augment antigen presentation and immune activation, advanced mRNA vaccine designs often integrate adjuvant functions. For instance, certain lipid-like materials, such as C1 LNPs, not only efficiently deliver mRNA but also act as TLR4 agonists. They promote dendritic cell maturation and the release of inflammatory cytokines such as IL-12, thereby creating a self-adjuvanting effect that synergistically boosts T cell activation along with antigen expression. (Zhang et al., 2021). Furthermore, combining mRNA vaccines with innate immune receptor agonists, such as STING agonists, enhances type I interferon and proinflammatory cytokine production, including TNF-α. This combination enhances dendritic cell antigen presentation and T cell activation more effectively, demonstrating potent antitumor activity in a tumor model (Zhang H. et al., 2026). In summary, mRNA vaccines synergistically activate CD8^+^ and CD4^+^ T cells through precise endogenous antigen synthesis and efficient dual MHC presentation pathways, laying the molecular foundation for robust and durable antitumor immunity.

### 3.2 Co-induction of cellular immunity, humoral immunity, and immune memory

A key advantage of mRNA cancer vaccines is the coordinated activation of cellular and humoral immunity, establishing a multi-layered anti-tumor defense network. Dendritic cells (DCs) act as the critical bridge between innate and adaptive immunity and play a central role in mRNA vaccine-induced responses (Wu R. et al., 2025). Upon uptake by DCs *via* delivery systems, the encoded tumor antigens are expressed intracellularly, processed, and presented to T cells to initiate adaptive immunity (Wu R. et al., 2025). This process effectively activates cytotoxic CD8^+^ T cells for direct tumor killing while engaging CD4^+^ helper T cells to provide essential help signals to both CD8^+^ T cells and B cells, coordinating the overall immune response (Wu R. et al., 2025). Concurrently, mRNA vaccines activate B cells to induce tumor antigen-specific antibody production, constituting the humoral response (Wu R. et al., 2025). Studies demonstrate that mRNA vaccines induce robust neoantigen-specific CD8^+^ T cell responses that effectively inhibit tumor growth across multiple murine models (Fan et al., 2024). Furthermore, optimizing delivery systems, such as using spleen-selective mRNA-LNPs, significantly enhances antigen-specific cellular immunity without triggering excessive inflammation (Gao et al., 2025). An important consideration is whether additional adjuvants, particularly Toll-like receptor (TLR) agonists, are required to elicit sufficient immune activation for cancer applications. Unlike prophylactic vaccines against infectious diseases, in which humoral immunity often serves as a key correlate of protection, therapeutic cancer vaccines generally depend more critically on robust CD8^+^ cytotoxic T-lymphocyte priming to eliminate antigen-expressing tumor cells and overcome tumor heterogeneity (Saxena et al., 2021). This requirement for effective CTL induction is further complicated by the immunosuppressive nature of many tumor microenvironments, particularly in poorly infiltrated or immunologically non-T cell-inflamed tumors (Chen and Mellman, 2017). TLR agonists, including poly(I:C) as a TLR3-associated agonist, TLR7/8 agonists, and CpG oligonucleotides as TLR9 ligands, can promote dendritic-cell maturation, enhance IL-12 and type I interferon production, and facilitate cross-priming of antigen-specific CD8^+^ T cells (Coffman et al., 2010). At the same time, mRNA and its delivery systems possess a degree of intrinsic adjuvant activity through innate immune sensing pathways, making this immunostimulatory effect an engineerable feature of the vaccine platform rather than an all-or-none requirement for external adjuvants (Pardi et al., 2018). This synergistic activation of cellular and humoral immunity enables mRNA vaccines to target tumors multidimensionally and overcome immune escape mechanisms.

The formation of memory T and B cells is critical for durable anti-tumor protection. Successful vaccination requires not only the clearance of existing tumors but also the establishment of long-term immune memory to prevent recurrence. Memory T-cell differentiation is supported by sustained antigen exposure and cytokine signaling, particularly interleukin-7 (IL-7) and IL-15, which promote the development and maintenance of central memory (Tcm) and effector memory (Tem) subsets. mRNA vaccines effectively induce long-lasting memory responses through their unique mechanisms. Preclinical studies show that mRNA vaccines formulated with lipopolyplexes (LPP) encoding tandem neoantigens completely prevent tumor development in prophylactic settings; the induced long-lived memory T cells protect mice against tumor rechallenge (Fan et al., 2024). This indicates the successful establishment of immune memory. The formation of memory B and T cells depends on antigen persistence and effective co-stimulatory signals. In the humoral arm, effective vaccine-induced CD4^+^ T-cell help, particularly through T follicular helper (Tfh) cells, supports germinal center reactions, where B cells undergo affinity maturation and clonal selection, ultimately giving rise to long-lived plasma cells and memory B cells. Novel mRNA vaccine designs facilitate this process by enhancing APC activation and antigen presentation efficiency. For instance, a manganese-based mRNA nanovaccine utilizing hybrid dendritic cell and bacterial membranes targets and resides in lymph nodes to sustain antigen presentation, thereby triggering potent T cell-mediated responses (He et al., 2025). Additionally, mRNA platforms co-delivering MHC-I and MHC-II restricted neoantigens significantly boost antigen-specific CD8^+^ T cell responses and reduce post-operative recurrence in colorectal cancer mouse models, suggesting robust memory formation alongside strong effector responses (Cho et al., 2025). In melanoma studies, combining mRNA vaccines with ICIs enhances therapeutic efficacy and may promote the establishment of long-term anti-tumor memory (Bafaloukos et al., 2023). Therefore, by optimizing antigen design, delivery systems, and combination strategies, mRNA vaccines hold the potential to induce potent and durable memory T and B cell responses, offering possibilities for long-term cancer control or cure.

### 3.3 Immune modulation and TME remodeling

Mechanistically, mRNA vaccines orchestrate antitumor immunity through three interconnected axes: efficient antigen presentation, amplification of tumor-specific adaptive immune responses, and reprogramming of the immunosuppressive TME. These processes are not independent; rather, the quality of antigen expression and presentation influences downstream T-cell priming, while the durability and functional impact of these responses ultimately depend on whether the local tumor milieu can support immune-cell infiltration and effector activity. A central challenge for mRNA cancer vaccines is overcoming the immunosuppressive state of the TME to reverse immune evasion. The TME is a complex ecosystem characterized by abundant regulatory T cells, M2-like tumor-associated macrophages, myeloid-derived suppressor cells, inhibitory cytokines, and aberrant metabolic pathways like enhanced glycolysis, which collectively impair effector immune cell function and sustain non–T cell–inflamed tumors (Li X. et al., 2022; Binnewies et al., 2018). While mRNA vaccines encoding tumor-specific antigens can induce potent antigen-specific CD8^+^ T cell responses, efficacy largely depends on remodeling the TME to enhance intratumoral T-cell infiltration and establish an immune-inflamed tumor phenotype. Studies suggest that mRNA vaccine design and delivery systems exert intrinsic immunomodulatory effects. In addition, mRNA platforms can be engineered to encode immunomodulatory payloads that act locally within the tumor or in antigen-presenting cells, thereby extending their function beyond antigen delivery alone (Hotz et al., 2021). For instance, certain nanovaccines enhance cancer immunotherapy by mimicking acute infection processes (Jin et al., 2024). Besides IFN-β, cytokines such as IL-12 represent attractive encoded payloads for mRNA-based immunotherapy because they promote Th1 polarization, enhance IFN-γ production, and activate NK cells and cytotoxic T lymphocytes (Trinchieri, 2003). Furthermore, intratumoral injection of mRNA vaccines, such as those encoding IFN-β, generates high local concentrations of immunostimulatory factors. This approach significantly increases intratumoral CD8^+^ T cell infiltration, elevates the CD8^+^/CD4^+^ T cell ratio, and repolarizes M2-like macrophages toward an anti-tumor M1 phenotype, effectively reprogramming the local immunosuppressive microenvironment (Kimura et al., 2025). Such local immune activation may also help weaken suppressive cellular networks within the TME, including Treg-associated restraint and myeloid-driven immunosuppression (Hotz et al., 2021). Such *in situ* vaccination strategies induce local anti-tumor immunity with potential abscopal effects. Thus, mRNA vaccines serve not only as antigen delivery vehicles but also as critical tools for modulating the TME and overcoming immune escape *via* their delivery systems, encoded immunomodulatory payloads, and administration routes.

The combination of mRNA vaccines with ICIs demonstrates significant synergy, representing an effective strategy to overcome TME immunosuppression and enhance therapeutic response. ICIs like anti-PD-1/PD-L1 and anti-CTLA-4 antibodies restore T-cell function by blocking inhibitory signals but often show limited efficacy against non–T-cell-inflamed tumors lacking pre-existing T-cell infiltration. This immune-excluded or non–T cell–inflamed phenotype is further reinforced by cellular and metabolically suppressive features of the TME, including lactate accumulation, adenosine signaling mediated by the CD39/CD73 axis, and activation of the indoleamine 2,3-dioxygenase (IDO)–kynurenine pathway, all of which can impair dendritic cell function and effector T-cell activity. mRNA vaccines address this limitation by priming and expanding antigen-specific T cells in draining lymph nodes, providing the essential effector cell pool required for ICI activity (Pati et al., 2025). Importantly, their contribution may extend beyond peripheral priming, as mRNA-based immunotherapies can also reshape the local TME through the induction of type I interferons or the expression of immune-stimulatory cytokine payloads (Hotz et al., 2021). Preclinical studies confirm this synergy. For example, in HPV-positive oropharyngeal squamous cell carcinoma models, combining an mRNA-LNP vaccine encoding HPV E7 with an anti-PD-1 antibody significantly enhanced the expansion of HPV-specific CD8^+^ T cells and maintained their anti-tumor function, thereby promoting tumor regression (Qiu et al., 2023). Similarly, in melanoma models, intratumoral injection of COVID-19-based mRNA vaccines combined with anti-PD-1 therapy delayed tumor growth and improved survival more effectively than monotherapy (Boehm et al., 2025). Mechanisms underlying this synergy are likely multifaceted: mRNA vaccines can induce local type I interferon responses, including IFN-β, which promote the expression of chemokines such as CXCL9 and CXCL10 to facilitate CD8^+^ T-cell recruitment and enhance MHC expression on tumor cells, thereby improving antigen visibility within the tumor. At the same time, ICIs release the brakes on these infiltrating T cells, enabling more effective tumor cell killing. Additionally, combination therapy facilitates antigen spreading, broadening the anti-tumor immune response (Li et al., 2022a). By increasing antigen-specific T-cell infiltration and inflammatory signaling, such combinations may also help counteract suppressive cellular and metabolic circuits within the TME, including Treg- and MDSC-associated immunosuppression, lactate-driven dendritic cell dysfunction, adenosine-mediated T-cell inhibition, and tolerance-promoting IDO–kynurenine signaling. In colorectal cancer models, neoantigen-targeted mRNA vaccines combined with ICIs synergistically inhibited tumor growth by inducing robust T-cell responses and promoting favorable TME alterations (Cho et al., 2025). These findings indicate that combining mRNA vaccines with ICIs simultaneously addresses the critical steps of “priming immune responses” and “releasing immune brakes,” while also promoting local immune remodeling within the TME, thereby offering a promising therapeutic avenue for refractory cancers, including pancreatic cancer and glioblastoma (Bloom et al., 2025; Strika et al., 2024).

The detailed mechanisms underlying mRNA-mediated anti-tumor immunity are listed in Table 2. Overall, the therapeutic power of mRNA cancer vaccines mainly comes from two combined mechanisms: CD8^+^ T cell-mediated targeted killing and reshaping the immunosuppressive tumor environment. By mimicking viral infection patterns, mRNA vaccines not only effectively promote the growth and infiltration of effector T cells through natural antigen presentation but also convert non-inflamed tumors into inflamed ones through innate immune activation and combination strategies. This dual ability, which both elicits strong effector responses and reverses immune suppression in the microenvironment, effectively combats tumor immune escape, laying the foundation for long-lasting tumor regression.

**TABLE 2:** mRNA-mediated anti-tumor mechanisms.

Immune activation mechanism	Core biological processes	Key significance for anti-tumor efficacy
1. Endogenous expression and dual MHC presentation	mRNA is delivered into the cytoplasm of antigen-presenting cells *via* lipid nanoparticles, translated into antigen proteins, and processed through the proteasome for MHC class I presentation. Antigens can also be secreted or cross-presented *via* MHC class II pathways.	Simultaneously activates CD8^+^ cytotoxic T cells and CD4^+^ helper T cells, generating a coordinated dual strike against tumor cells.
2. Innate immune self-adjuvant effects	mRNA sequences or delivery vectors engage pattern recognition receptors such as TLR4 and STING, triggering the production of type I interferons and pro-inflammatory cytokines.	Promotes dendritic cell maturation, enhances antigen presentation, and supplies critical danger signals and co-stimulatory molecules for effective priming of adaptive immunity.
3. Induction of potent cellular immunity	Massive expansion of neoantigen-specific CD8^+^ T cells, followed by their infiltration into tumor tissues and specific recognition and lysis of antigen-expressing cancer cells.	Serves as the central effector mechanism for solid tumor clearance, offering high specificity and the capacity to drive direct tumor regression.
4. Synergy with humoral immunity	With help from CD4^+^ T cells, B cells are activated and differentiate into plasma cells, producing high-affinity antibodies targeting tumor surface antigens.	Enables antibody-dependent cellular cytotoxicity and establishes a multi-layered immune defense that complements cellular immunity.
5. Establishment of long-term memory	Sustained antigen stimulation and retention in lymph nodes induce the differentiation of central memory T cells, effector memory T cells, and memory B cells.	Provides durable immune surveillance, enabling rapid and robust responses upon tumor recurrence or metastasis, thereby preventing disease progression.
6. Tumor microenvironment remodeling and reprogramming	Induces secretion of IFN-γ and IFN-β, repolarizes M2-like macrophages toward an M1-like phenotype, and synergizes with immune checkpoint inhibitors to relieve immune suppression.	Converts non-T cell-inflamed tumors into T cell-inflamed tumors, reversing immune escape and improving response rates to combination therapies.

ADCC, antibody-dependent cellular cytotoxicity; APC, antigen-presenting cell; CD4^+^, cluster of differentiation 4 positive; CD8^+^, cluster of differentiation 8 positive; CTL, cytotoxic T lymphocyte; DC, dendritic cell; ICI, immune checkpoint inhibitor; IFN, interferon; LNP, lipid nanoparticle; MHC, major histocompatibility complex; STING, stimulator of interferon genes; Tcm, central memory T cell; Tem, effector memory T cell; TLR, Toll-like receptor; TME, tumor microenvironment.

## 4 mRNA vaccine delivery systems

Given that antigen expression, cross-presentation, and innate immune sensing all depend on efficient cytosolic delivery, the design of mRNA delivery systems fundamentally determines the magnitude and quality of antitumor immunity. Delivery vehicles are therefore not merely passive carriers that protect mRNA from degradation; they actively shape biodistribution, cellular uptake, endosomal escape, and immune activation, thereby influencing how effectively vaccine-encoded antigens are translated and recognized by the host immune system.

### 4.1 LNP technology

LNPs are the cornerstone of mRNA vaccine delivery, typically comprising four components: ionizable cationic lipids, phospholipids, cholesterol, and PEGylated lipids (Wu S. et al., 2024). Among these, ionizable lipids are critical; their pH-dependent charge enables efficient mRNA encapsulation during formulation while minimizing systemic toxicity at physiological pH (Paroor et al., 2025). Upon acidification in endosomes, these lipids become protonated, promoting electrostatic interactions with anionic endosomal lipids, destabilizing the membrane, and forming non-bilayer hexagonal phases that facilitate cytosolic release of mRNA. PEG-lipids reside on the particle surface, providing steric stabilization to enhance colloidal stability, prolong circulation time, and modulate cellular uptake (Wang M. M. et al., 2023). In biological fluids, the formation of a protein corona, particularly the adsorption of apolipoprotein E (ApoE), can further shape LNP biodistribution and promote hepatic uptake. For manufacturing, microfluidic mixing has emerged as the industry standard. By controlling fluid dynamics within microchannels, this technique drives the rapid self-assembly of components into uniform nanoparticles (60–150 nm) with high reproducibility, effectively resolving the scalability limitations of traditional ethanol injection (Lopes et al., 2022; Wu W. et al., 2025). LNP-based mRNA vaccines have demonstrated remarkable efficacy, exemplified by the SARS-CoV-2 vaccines BNT162b2 and mRNA-1273, highlighting the platform’s potential in infectious disease prevention (Kon et al., 2022). This success has significantly accelerated the exploration of LNP-mRNA technology in oncology, with numerous vaccines targeting tumor-associated antigens or neoantigens advancing into preclinical and clinical stages for cancer immunotherapy (Wu Y. et al., 2024; Ramadan et al., 2024).

The LNP delivery system offers multiple advantages. The modular design of LNPs permits rational optimization of lipid components to balance potency, stability, and toxicity (Kon et al., 2022). Screening ionizable lipid libraries enables targeted delivery to lymphoid tissues like the spleen, which is crucial for activating robust cellular immunity (Yoo et al., 2025; Zhang H. et al., 2025). Furthermore, LNPs protect mRNA from serum degradation and facilitate endocytic uptake by antigen-presenting cells, ensuring cytoplasmic antigen translation (Zhang X. et al., 2024; Malburet et al., 2023). However, intrinsic immunogenicity remains a challenge; LNP components, particularly ionizable lipids, can trigger innate immune activation and pro-inflammatory cytokine release like IL-6 and TNF-α, leading to systemic side effects (Igyártó et al., 2021; Kawaguchi et al., 2025). Notably, even empty LNPs can activate monocytes and dendritic cell subsets (Zelkoski et al., 2026). While this adjuvant effect aids immune priming, excessive inflammation poses safety risks and may suppress mRNA translation (Gao et al., 2025; Yang et al., 2024). Mechanistically, excessive innate sensing can activate protein kinase R (PKR) and induce eIF2α phosphorylation, thereby attenuating mRNA translation efficiency; activation of the OAS–RNase L pathway may further accelerate RNA degradation and limit protein production. Consequently, research now focuses on modulating reactogenicity through formulation optimization. Key strategies include developing biodegradable ionizable lipids (Yoo et al., 2025), adjusting the chain length and molar ratio of PEG-lipids to alter surface properties, substituting cholesterol with phytosterols or modifying phospholipid headgroups and tail structures (Kawaguchi et al., 2025), or employing non-cationic systems to mitigate charge-induced toxicity (Wang C. et al., 2023). These optimizations aim to achieve “decoupling”—maximizing vaccine-induced cellular immunity while minimizing unnecessary inflammatory side effects—thereby expanding the therapeutic window and safety profile of mRNA-LNP cancer vaccines (Gao et al., 2025). Representative LNP-based mRNA cancer vaccines in clinical development are summarized in Table 3.

**TABLE 3:** mRNA vaccines prepared from LNPs of different categories.

Vaccine category	Drug name	Encoded content	Target cancer type	Developer	Clinical stage	Technical features
Off-the-shelf cancer vaccines	BNT111	NY-ESO-1, MAGE-A3, tyrosinase, TPTE	Melanoma	BioNTech	Phase II	Fixed multi-antigen combination with broad coverage
​	BNT113	HPV16 E6/E7 oncoproteins	HPV16-positive head and neck squamous cell carcinoma	BioNTech	Phase II	Targeting virus-associated cancers
​	BNT116	Six NSCLC-related tumor-associated antigens	Non-small cell lung cancer	BioNTech/Regeneron	Phase I/II	Multi-antigen synergistic strategy
​	mRNA-4359	Ido1 and PD-L1 immune checkpoints	Melanoma, non-small cell lung cancer	Moderna	Phase I/II	Checkpoint vaccine mechanism
​	CVGBM	Eight peptide fragments from four GBM-associated antigens	Glioblastoma (MGMT-unmethylated)	CureVac	Phase I	Targeted patient population
​	BNT112	Five prostate cancer-specific antigens	Prostate cancer	BioNTech	Phase I	RNA-LPX platform
Individualized neoantigen vaccines	mRNA-4157 (V940)	Up to 34 patient-specific neoantigens	Melanoma	Moderna/Merck	Phase III	Combined with PD-1 inhibitors showing significant efficacy
​	BNT-122 (iNeST)	Approximately 20 patient-specific neoantigens	Pancreatic cancer, colorectal cancer, melanoma	BioNTech/Genentech	Phase I/II	Precision customization based on patient-specific mutations
​	NeoPol-mL242	Personalized neoantigens	Hepatocellular carcinoma	Wu Y. et al. (2025)	Preclinical	Spleen-targeting L242-20Lipo delivery system
Mutation-specific or immunomodulatory vaccines	V941 (mRNA-5671)	KRAS mutations (G12D/V, G13D, G12C)	Pancreatic, colorectal, and non-small cell lung cancer	Merck/Moderna	Phase I	Targeting specific KRAS mutations
​	mRNA-2752	OX40L, IL-23, IL-36γ	Relapsed/refractory solid tumors	Moderna	Phase I	Intratumoral injection for TME remodeling
​	BCMA-mRNA	BCMA antigen	Multiple myeloma	Dutta et al. (2025)	Preclinical	Encapsulated with Poly(I:C) For enhanced immune response
Advanced delivery and technology optimization	pNB-LNP	HER2-targeted delivery platform	Multiple cancer models	Zhu et al. (2026)	Preclinical	PEG-free palmitoylated nanobody for active targeting
​	LNP 5097	Model antigen	Cancer vaccine models	Yoo et al. (2025)	Preclinical	Organ-selective delivery with vitamin B5 derivative
​	MUC1-NLE	MUC1 mRNA combined with CTLA-4 siRNA	Triple-negative breast cancer	Monfaredan et al. (2025)	Preclinical	Nano-lipid exosome system for co-delivery
​	α-lactalbumin mRNA-LNP	α-Lactalbumin	Triple-negative breast cancer	He et al. (2024)	Preclinical	Combined with surgery to suppress progression and metastasis

BCMA, B-cell maturation antigen; CTLA-4, cytotoxic T-lymphocyte-associated protein 4; GBM, glioblastoma multiforme; HER2, human epidermal growth factor receptor 2; HPV, human papillomavirus; IDO1, indoleamine 2,3-dioxygenase 1; IL, interleukin; KRAS, kirsten rat sarcoma viral oncogene homolog; LNP, lipid nanoparticle; MAGE-A3, melanoma-associated antigen 3; MGMT, O6-methylguanine-DNA, methyltransferase; NSCLC, non-small cell lung cancer; NY-ESO-1, New York esophageal squamous cell carcinoma 1; OX40L, OX40 ligand; PD-1, programmed death-1; PD-L1, programmed death-ligand 1; siRNA, small interfering RNA; TAA, tumor-associated antigen; TME, tumor microenvironment; TPTE, transmembrane phosphatase with tensin homology.

### 4.2 Polymer nanoparticles and novel delivery vectors

Polymer nanoparticles serve as versatile non-viral vectors in mRNA vaccine delivery due to their tunable chemical structures, biocompatibility, and ease of functionalization (Yang et al., 2023). Compared with lipid systems, polymeric vectors offer distinct advantages in modulating pharmacokinetics, engineering biodistribution, and enabling tissue-specific delivery (Yang et al., 2023). In terms of mRNA encapsulation, LNPs achieve encapsulation efficiencies typically exceeding 85% through microfluidic self-assembly, whereas polymeric carriers rely on electrostatic complexation, whose efficiency is more sensitive to formulation conditions and tends to yield greater batch-to-batch variability (Zhang et al., 2022). Rather than being viewed solely through the lens of material composition, these emerging delivery systems can also be understood functionally in terms of how they enhance antitumor immunity.

Lymph node-targeting systems are particularly attractive because efficient antigen delivery to lymphoid tissues is critical for dendritic cell priming and T-cell activation. For instance, tuning the PEGylation degree of polyaspartic acid nanoparticles enables targeted lung expression following intravenous injection or localized expression after intramuscular administration (Park et al., 2022). In parallel, site-specific polymer engineering has enabled the preferential localization of mRNA expression and immune activation in tumor-draining lymph nodes, thereby maximizing vaccine efficacy while minimizing systemic toxicity (Guo et al., 2025). By comparison, LNP-based DC targeting has been advanced through surface conjugation of DC-specific antibodies; for instance, LNPs functionalized with anti-CLEC9A antibodies achieved extrahepatic delivery to the spleen and lymph nodes, resulting in approximately 50% greater tumor growth inhibition than unconjugated LNPs, while CD40-targeted PLGA nanoparticles showed superior DC internalization compared with DEC-205 or CD11c-targeted counterparts (Zhang Z. et al., 2026). Furthermore, a library of STING-activating polymers (PD) composed of tertiary amines and biodegradable alkyl chains has been engineered for lymphatic delivery. Among these, the PD18 variant, which contains 18 tertiary amines, achieved an optimal balance between immune activity and tolerance (Zhang M. et al., 2025). Studies demonstrate that PD18 nanoparticles loaded with antigen mRNA significantly expand antigen-specific CD8^+^ T cells and establish long-term immune memory following subcutaneous injection. Notably, this platform exhibited superior antitumor efficacy compared with the conventional adjuvant 2′3′-cGAMP in both prophylactic and therapeutic models, highlighting its potential to maximize potency with minimal systemic toxicity (Zhang M. et al., 2025).

Intrinsic adjuvant polymers provide a second functional advantage by coupling mRNA transport with immune stimulation. To enhance vaccine immunogenicity, researchers have developed polymers that integrate delivery functions with built-in adjuvant activity. For example, biodegradable polyβ-amino ester (PBAE) has been shown to efficiently transfect dendritic cells (DCs) and induce their maturation (Fornaguera et al., 2021). Likewise, nanoparticles modified with polyguanidinium thioctic acid (POctS) facilitate efficient mRNA delivery while simultaneously activating the STING pathway to boost innate immunity (Guo et al., 2025). Such multifunctional polymers not only protect mRNA and improve cytosolic delivery, but also promote local immune activation, offering a strategy to enhance potency without relying exclusively on separately administered adjuvants. By comparison, LNP-based systems can similarly couple payload delivery with immune modulation, for instance, QTsome LNPs encapsulating anti-miR-21 achieved over 80% tumor growth inhibition alongside marked CD8^+^ T cell and M1 macrophage expansion in the TME (Zhang Z. et al., 2023). Though this immune activation is primarily driven by ionizable lipid-TLR4 interactions, which promote DC maturation and cytokine release but carry the risk of suppressing mRNA translation when innate activation is excessive, a trade-off that polymeric systems with tunable immunostimulatory profiles may help circumvent (Luo et al., 2025).

TME-responsive systems have emerged to address the immunosuppressive and spatially heterogeneous nature of solid tumors. A notable innovation, the PSB@Nb1.33C/mRNA system, leverages the light-driven locomotion and hypoxia-taxis of photosynthetic bacteria (PSB) to deliver mRNA-loaded 2D iMXene nanosheets precisely into the tumor core (Zhang S. et al., 2024). This design exploits TME features, particularly hypoxia, to enhance intratumoral penetration and local payload accumulation. By enhancing delivery into otherwise poorly accessible tumor regions, such systems may help improve antigen expression and immune activation within immunologically “cold” or structurally complex tumors (Zhang S. et al., 2024).

Stimuli-responsive systems further extend this concept by enabling controlled release or activation in response to external or local triggers. In the PSB@Nb1.33C/mRNA platform, near-infrared irradiation activates the photothermal effect of iMXene, which triggers immunogenic cell death (ICD) and results in the substantial release of the mRNA payload; the released mRNA is then translated into antigens that boost downstream immune responses. (Zhang S. et al., 2024). Termed “photo-immunogenic gene cancer therapy,” this strategy effectively inhibited primary tumor growth, postoperative metastasis, and distant tumors (Zhang S. et al., 2024). In addition, hydrogel-based platforms show significant promise as controllable delivery systems; for example, injectable polymer-nanoparticle hydrogels can recruit key immune cells to form a tunable *in vivo* immune microenvironment, significantly extending the durability and breadth of humoral responses to mRNA vaccines (Meany et al., 2025). Together, these systems provide spatial and temporal control over antigen release and immune activation, which may be especially valuable for improving therapeutic precision and limiting off-target effects.

Beyond traditional LNPs, diverse platforms, including polymer nanoparticles such as PLA, PBAE, and lipid-polymer hybrids, alongside functionalized nanocarriers like LCP, CLAN, and spleen-targeting or bio-responsive LNPs, constitute important avenues for mRNA delivery. By employing distinct material combinations, targeting ligands including mannose or specific peptides, and intelligent response mechanisms, these systems aim to optimize mRNA stability, specific cellular uptake, antigen presentation efficiency, and immune activation. Although currently predominant in preclinical development, these platforms have demonstrated significant potential in inducing potent antitumor immunity across various murine tumor models.

### 4.3 Controlled activation and targeted delivery strategies

Developing controlled activation and targeted delivery strategies is essential for optimizing mRNA vaccines in cancer therapy. These approaches aim to precisely regulate immune activation and enrich for target tissues or cells, thereby enhancing therapeutic efficacy while mitigating systemic toxicity. Current research primarily focuses on using physical stimuli to activate regions and on the rational design of nanocarriers to achieve targeted delivery.

Given that systemic administration may trigger nonspecific inflammation, achieving regional release *via* physical or chemical stimuli has become a key research focus. While direct ultrasound activation of LNPs is less explored, the concept of controlled release is well-established in nanomedicine. For instance, near-infrared light-driven nanomotors have been developed to utilize high hydrogen sulfide levels in the TME as both a chemoattractant and a response trigger for targeted release (Wu L. et al., 2025). This responsive strategy offers a blueprint for regional mRNA vaccine activation. By encapsulating mRNA in carriers responsive to endogenous signals such as acidic pH and enzymes, or external physical fields including light, magnetism, and ultrasound, vaccines can be released locally at the tumor site (Eslami et al., 2025). This on-demand activation confines immune stimulation to the target region, effectively circumventing systemic adverse effects.

Efficient delivery relies on precise targeting of the lymphatic system or antigen-presenting cells. Passive targeting largely relies on the size effect of nanoparticles. Studies indicate that LNPs ranging from 200 to 500 nm tend to accumulate in the spleen and are efficiently internalized by splenic DCs (Sasaki et al., 2022). Precise regulation of the alkyl chain length and molar ratio of PEGylated lipids alters LNP physicochemical properties and protein corona composition, redirecting biodistribution from the liver to the spleen to facilitate spleen-targeted mRNA delivery, a process critical for activating adaptive immunity (Liu S. et al., 2026). Active targeting is achieved through specific ligand modification. For example, the manganese-coordinated multivalent aptamer system, known as COMPASS, employs rolling circle amplification to construct scaffolds bearing multiple targeting aptamers, enabling the co-delivery of mRNA and manganese ions to lymph node dendritic cells for enhanced immune activation (Liu X. et al., 2026). Similarly, a novel mRNA nanovaccine (HM@Mn_3_O_4_-mRNA) coated with a hybrid membrane derived from DCs and bacteria demonstrated the ability to target DCs and reside within lymph nodes to sustain antigen presentation (He et al., 2025). Furthermore, utilizing tumor cell-derived exosomes as a vaccine platform has demonstrated dual targeting capabilities to lymph nodes and the brain, inducing potent protective immunity in glioblastoma models (Zou et al., 2025). The development of these targeting strategies significantly advances the precision and potency of mRNA vaccines in tumor immunotherapy. Despite these advances, achieving efficient cytosolic delivery in target cells while minimizing off-target distribution and systemic reactogenicity remains a central challenge for translational application. Collectively, delivery system design not only governs biodistribution and antigen expression kinetics but also shapes innate sensing thresholds and adaptive immune polarization, underscoring the tight integration of biomaterials engineering and immunological programming in determining the therapeutic efficacy of mRNA cancer vaccines.

### 4.4 Current therapeutic approaches: combination strategies, immunologic adjuvants, and personalized immunotherapy

The role of mRNA vaccines in oncology has expanded beyond monotherapy. Increasing evidence supports their integration with chemotherapy, radiotherapy, and ICIs, where synergistic effects are emerging. Preclinical and early clinical studies show that mRNA vaccines activate host immunity, enhance antigen presentation, and amplify tumor-specific T cell responses, thereby establishing a rational foundation for combination therapy (Lorentzen et al., 2022). Among combination strategies, pairing mRNA vaccines with PD-1/PD-L1 or CTLA-4 inhibitors has shown particular promise. In melanoma, the combination of mRNA vaccination with ICIs has yielded encouraging outcomes (Bidram et al., 2021). The KEYNOTE-942 trial provided the first clinical evidence that the personalized mRNA vaccine mRNA-4157/V940 combined with pembrolizumab significantly improved patient outcomes (Bafaloukos et al., 2023; Han et al., 2024). Mechanistically, mRNA vaccines expand tumor-specific T-cell clones, whereas ICIs restore TCR signaling by blocking PD-1–SHP2-mediated inhibitory cascades or CTLA-4-dependent costimulatory competition, thereby sustaining effector function within the TME and generating synergistic antitumor activity (Fan et al., 2024). Similar findings were reported in a gastric cancer peritoneal metastasis model, where a neoantigen mRNA-LNP vaccine combined with anti-PD-1 therapy elicited stronger neoantigen-specific CD8^+^ T cell responses and achieved tumor regression, outperforming monotherapy (Nagaoka et al., 2025). Beyond ICIs, mRNA vaccines are being incorporated into multimodal regimens. Chemotherapy and radiotherapy can further enhance vaccine responsiveness by inducing immunogenic cell death, characterized by calreticulin exposure, HMGB1 release, and ATP secretion, which promote dendritic cell recruitment and cross-priming (Azadi et al., 2021). Local radiofrequency ablation combined with a neoantigen peptide vaccine demonstrated synergistic effects in both patients and murine models and further benefited from PD-1 blockade (Shou et al., 2022). Emerging strategies also include co-delivery of immunostimulatory adjuvants such as STING agonists or Toll-like receptor agonists, including TLR3, TLR7, and TLR8 ligands, as well as cytokine support with IL-7, IL-12, or IL-15. In this context, IL-15 supports the maintenance of memory CD8^+^ T cells, whereas IL-12 promotes Th1 polarization and IFN-γ production, thereby reinforcing cellular immunity. Another rapidly developing direction is the convergence of mRNA platforms with CAR-T cell therapy. In this setting, mRNA does not function solely as a vaccine antigen source, but can also be used to selectively expand or generate CAR-T cells *in vivo*. A novel concept, CARVac, has been proposed, in which a nanoparticulate RNA vaccine encoding the CAR target antigen claudin 6 (CLDN6) was delivered to antigen-presenting cells, thereby promoting cognate antigen presentation, selective expansion of CLDN6-specific CAR-T cells, and enhanced tumor regression in preclinical solid tumor models (Reinhard et al., 2020). This approach has since advanced to clinical testing in the phase 1/2 BNT211-01 trial, where CLDN6-specific CAR-T cells combined with an amplifying RNA vaccine demonstrated manageable toxicity and encouraging preliminary antitumor activity in patients with relapsed or refractory solid tumors (Mackensen et al., 2023). Beyond adoptive transfer-based boosting, mRNA-LNP technology may also enable the direct generation of CAR-T cells *in vivo*. It was shown that targeted delivery of CAR-encoding mRNA can transiently reprogram endogenous T cells *in vivo*, providing proof of concept for bypassing conventional *ex vivo* manufacturing (Rurik et al., 2022). Although this strategy remains at an early stage and was initially demonstrated outside oncology, it highlights a rapidly advancing interface between mRNA delivery systems and cell-based immunotherapy. In addition, integration with oncolytic viruses or CRISPR-based gene editing is under active investigation, including strategies to restore β2-microglobulin-dependent antigen presentation, reduce PD-L1 expression, or correct defects in JAK/IFN signaling, reflecting a shift toward coordinated, multi-layered immunotherapy platforms. Collectively, these findings position mRNA-based combination regimens as a central direction in future cancer immunotherapy.

One of the most transformative advantages of mRNA technology lies in personalized vaccine design guided by tumor genomic profiling. Unlike conventional vaccines targeting shared tumor-associated antigens, personalized mRNA vaccines encode neoantigens derived from tumor-specific mutations. These antigens are uniquely expressed in malignant cells, enabling highly specific immune responses with minimal off-target toxicity (Wu A. C. et al., 2025). The design process begins with whole-exome or transcriptome sequencing of patient tumor samples, followed by bioinformatic prioritization of immunogenic neoepitopes. Contemporary prediction pipelines integrate parameters such as MHC-binding affinity, proteasomal cleavage likelihood, and, where feasible, immunopeptidomic validation to refine neoantigen selection (Imani et al., 2025). Selected neoantigens are encoded into synthetic mRNA constructs and formulated using efficient delivery systems such as lipid nanoparticles (Yao et al., 2024). This strategy reconciles personalization with scalability, allowing individualized treatment while enabling HLA-informed epitope selection tailored to individual patients and maintaining relatively rapid, potentially cost-controlled production (Sayour et al., 2024). Clinical progress has been reported in multiple malignancies, including multiple myeloma, colorectal cancer, and glioblastoma (Cho et al., 2025; Trivedi et al., 2024; Feng et al., 2025). For example, a tandem neoantigen mRNA vaccine delivered by a lipid-polyplex (LPP) system induced robust neoantigen-specific CD8^+^ T cell responses in preclinical models and generated meaningful immune and clinical responses in two patients (Fan et al., 2024). Technological convergence further strengthens personalized immunotherapy. CRISPR-based gene editing may further sensitize tumors by restoring antigen presentation machinery likeβ2-microglobulin, knocking out immunosuppressive molecules such as PD-L1, or correcting defects in JAK pathway signaling, thereby enhancing subsequent responses to neoantigen mRNA vaccines[98]. (Tanriverdi, 2025). Artificial intelligence has improved the accuracy of neoantigen prediction and optimized mRNA sequence design and LNP formulation, increasing stability and immunogenicity (Imani et al., 2025; Di Salvatore et al., 2025). Including deep learning models such as EDGE and MARIA that jointly predict MHC binding affinity, antigen processing, and T-cell recognition probability (Zhang Z. et al., 2026). Despite challenges related to neoantigen prediction accuracy, manufacturing timelines, and regulatory pathways, personalized mRNA vaccines combine safety, potent immunogenicity, and rapid development cycles. They are emerging as a pivotal platform for precision oncology and are redefining therapeutic paradigms across multiple cancer types (Feng et al., 2025; Ibragimova et al., 2025). However, optimal treatment sequencing, dosing strategies, and biomarker-guided patient selection remain to be fully defined. Collectively, the integration of mRNA vaccination with ICIs, cytotoxic therapies, and genomic-guided personalization represents a rational convergence of immunologic priming, microenvironmental modulation, and precision oncology.

## 5 Immune response assessment of mRNA vaccines in cancer patients

Cancer and anticancer treatment substantially influence immune responses to mRNA vaccination, with the degree of impairment varying according to tumor type, treatment modality, and vaccination timing. Overall, mRNA vaccines show favorable tolerability in oncology populations, although vaccine-induced humoral responses are generally weaker than those observed in healthy individuals. Treatment modality is a major determinant of response: patients with solid tumors tend to develop stronger and more sustained antibody responses than those with hematologic malignancies, whereas B-cell–depleting therapies such as anti-CD20 monoclonal antibodies exert the most profound suppressive effects by disrupting germinal center formation and impairing affinity maturation, thereby compromising durable antibody production (Figueiredo et al., 2021). Chemotherapy also attenuates early humoral responses, but available evidence suggests that this effect may reflect delayed rather than permanently abolished immunity, as antibody levels can recover over time after treatment completion (Shroff et al., 2021). Booster immunization is therefore particularly important in this setting; a third dose significantly increases seropositivity rates and antibody titers across cancer subgroups, while adverse events remain predominantly mild and transient (Giuliano et al., 2023). Importantly, antibody titers alone do not fully capture vaccine-induced protection in patients with cancer. Even in individuals with severely impaired humoral immunity following B-cell–depleting therapy, antigen-specific CD4^+^ and CD8^+^ T-cell responses can still be detected, suggesting that cellular immunity may partially compensate for humoral deficiencies (Shroff et al., 2021). Collectively, these findings indicate that mRNA vaccine immunogenicity in cancer patients is heterogeneous but potentially modifiable, and that optimizing vaccination timing relative to systemic therapy together with appropriate booster strategies may improve protective immune responses in oncology populations.

## 6 Transformational application and clinical progress of mRNA vaccines

### 6.1 Landscape of clinical trials

A total of 60 clinical trials were identified and analyzed through the ClinicalTrials.gov database, of which 55 (91.7%) were classified as interventional trials and 5 (8.3%) as observational trials, indicating that this field remains predominantly focused on therapeutic and interventional exploration. Specifically, 21 trials (35%) are currently in the recruitment phase, while 16 trials (26.7%) are in the not yet recruiting stage, together accounting for the majority of ongoing studies. In contrast, only seven trials (11.7%) have been completed, reflecting a relatively limited number of mature investigations. The development status of these trials suggests an early yet active phase. A small proportion of studies were discontinued: three trials (5%) were terminated, and 1 trial (1.7%) was withdrawn. The geographical distribution of the included clinical trials is summarized in Table 4, highlighting China’s dominant contribution to this research area. Among research institutions, 45 distinct institutions or enterprises participated in these studies, reflecting broad engagement. Among them, *West China Hospital* (7 trials), *Ruijin Hospita*l (4 trials), and *Sir Run Run Shaw Hospital* (3 trials) emerged as the leading initiators. In parallel, the involvement of international biotechnology companies, such as CureVac, further underscores a research landscape driven jointly by academic institutions and industry partners. Regarding research content, the included trials span a wide range of solid tumors and central nervous system malignancies. Notably, studies targeting solid tumors constituted the most prevalent disease category (n = 14), underscoring the central role of mRNA vaccines in the exploration of broad-spectrum solid tumor therapies. In addition, studies on blood malignancies, such as lymphoma (n = 4), and on refractory tumors, such as glioblastoma (NCT05938387), although small in number, underscore exploratory efforts across various types of malignant tumors. Furthermore, a subset of studies incorporated COVID-19 vaccination or infection-related contexts. Collectively, these trials assessed not only the safety, tolerability, and preliminary efficacy of tumor-associated mRNA vaccines but also evaluated post-vaccination immune responses and imaging alterations in cancer patients.

**TABLE 4:** Clinical trials of mRNA vaccines and related immunotherapies in oncology and virus-associated conditions.

NCT number	Study title	Study status	Conditions	Sponsor	Study type
NCT05714748	Application of mRNA immunotherapy technology in Epstein-Barr virus-related refractory malignant tumors	ACTIVE_NOT_RECRUITING	Malignant tumors	West China Hospital	INTERVENTIONAL
NCT05938387	Safety and tolerability of CVGBM in adults with newly diagnosed MGMT-unmethylated glioblastoma or Astrocytoma	ACTIVE_NOT_RECRUITING	Glioblastoma	CureVac	INTERVENTIONAL
NCT05556720	Bringing optimised COVID-19 vaccine schedules to ImmunoCompromised populations (BOOST-IC): An adaptive randomised controlled clinical trial	ACTIVE_NOT_RECRUITING	HIV|organ transplantation|lymphoma, non-hodgkin|chronic lymphocytic leukemia|multiple myeloma|COVID-19 vaccines	Monash University	INTERVENTIONAL
NCT05270967	FDG (fluorodeoxyglucose) findings after COVID-19 vaccination	COMPLETED	Vaccine reaction|oncology	Kocaeli University	OBSERVATIONAL
NCT04872738	Patient Experiences with the COVID-19 vaccination after breast cancer treatment	COMPLETED	Breast cancer related lymphedema|COVID-19	Massachusetts General Hospital	OBSERVATIONAL
NCT03164772	Phase 1/2 study of combination immunotherapy and messenger ribonucleic acid (mRNA) vaccine in subjects with NSCLC	COMPLETED	Metastatic non-small cell lung cancer|NSCLC	Ludwig Institute for Cancer Research	INTERVENTIONAL
NCT00204516	Vaccination with tumor mRNA in metastatic melanoma - fixed combination versus individual selection of targeted antigens	COMPLETED	Malignant melanoma	University Hospital Tuebingen	INTERVENTIONAL
NCT05028374	COVID-19 VAX booster dosing in patients with hematologic malignancies	COMPLETED	Multiple myeloma|AL amyloidosis|chronic lymphocytic leukemia	Barbara Ann Karmanos Cancer Institute	INTERVENTIONAL
NCT04969601	Anti-COVID-19 vaccine in children with acute leukemia and their siblings	COMPLETED	Acute leukemia|acute lymphoblastic leukemia|acute myeloid leukemia	Assistance Publique - H么Pitaux de Paris	INTERVENTIONAL
NCT04918940	Efficacy of COVID-19 vaccination in Patientstreated with Anti-CD20 for follicular lymphoma or mantle cell lymphoma	COMPLETED	Follicular lymphoma|mantle cell lymphoma	Centre Henri Becquerel	INTERVENTIONAL
NCT07245901	Safety and tolerability of a mRNA vaccine encoding tumor-specific circular RNA antigens in combination with anti-PD-1 monoclonal antibody in patients with advanced solid tumors	NOT_YET_RECRUITING	Solid tumor	Sun Yat-sen memorial Hospital of sun Yat-sen University	INTERVENTIONAL
NCT07101536	The application of mRNA immunotherapy technology in refractory malignancies associated with Epstein-Barr virus (EBV)	NOT_YET_RECRUITING	EBV-associated tumors	West China Hospital	INTERVENTIONAL
NCT07363369	Exploratory clinical study of FAP mRNA vaccine in patients with advanced malignant solid tumors	NOT_YET_RECRUITING	Advanced malignant solid tumors	West China Hospital	INTERVENTIONAL
NCT05949775	Clinical study of mRNA vaccine in patients with advanced malignant solid tumors	NOT_YET_RECRUITING	Advanced malignant solid tumors	Stemirna therapeutics	INTERVENTIONAL
NCT07334574	Clinical study of XP-006 mRNA vaccine for R/R B-NHL	NOT_YET_RECRUITING	B-cell Non-Hodgkin’s lymphoma (B-NHL)	Ruijin Hospital	INTERVENTIONAL
NCT06788600	Mechanistic study of EBV mRNA vaccine (WGc-043) in EBV-positive relapsed/Refractory lymphoma	NOT_YET_RECRUITING	Epstein-Barr virus associated lymphoma	Ruijin Hospital	OBSERVATIONAL
NCT06932861	Exploratory study of personalized mRNA vaccine in patients with refractory rhabdomyosarcoma	NOT_YET_RECRUITING	Rhabdomyosarcoma	Xinxin Zhang	INTERVENTIONAL
NCT07073183	Safety and tolerability of CVHNLC plus pembrolizumab in patients with squamous non small-cell lung cancer (sqNSCLC)	NOT_YET_RECRUITING	Squamous NSCLC	CureVac	INTERVENTIONAL
NCT06685653	Personalized neoantigen MRNA vaccine combined with Adebrelimab in non-small cell lung cancer patients	NOT_YET_RECRUITING	NSCLC	Nanjing tianyinshan Hospital	INTERVENTIONAL
NCT06913218	A study of mRNA vaccines AK154 monotherapy or in combination with AK104/AK112, and sequential mFOLFIRINOX in surgically resected PDAC	NOT_YET_RECRUITING	Pancreas cancer|pancreas cancer, duct cell adenocarcinoma	Akeso	INTERVENTIONAL
NCT06273553	A study in subjects with human papillomavirus 16 or 18 associated cervical intraepithelial neoplasia grade 2 or 3	NOT_YET_RECRUITING	Human papillomavirus associated intraepithelial neoplasia|cervical intraepithelial neoplasia grade 2/3|Human papillomavirus type 16 Infection|Human papillomavirus type 18 infection	RinuaGene biotechnology co., Ltd.	INTERVENTIONAL
NCT06156267	Study of personalized tumour vaccines and a PD-L1 Blocker in patients with surgically resected pancreatic adenocarcino	NOT_YET_RECRUITING	Pancreatic cancer	Fudan University	INTERVENTIONAL
NCT07349836	Application of mRNA immunotherapy technology in EB virus related diseases	NOT_YET_RECRUITING	EBV-associated tumors	Xinqiao Hospital of Chongqing	INTERVENTIONAL
NCT07341321	SARS-CoV-2 mRNA vaccination in patients with hepatocellular carcinoma treated with immune checkpoint inhibitors	NOT_YET_RECRUITING	mRNA vaccination|hepatocellular carcinoma|checkpoint inhibitor	Medical University of graz	OBSERVATIONAL
NCT07077369	WGc-0201 plus tislelizumab in HCC with high risk of recurrence and metastasis after radical therapy	NOT_YET_RECRUITING	Hepatocellular carcinoma (HCC)	West China Hospital	INTERVENTIONAL
NCT06735508	MRNA neoantigen vaccine in non-small cell lung cancer	NOT_YET_RECRUITING	NSCLC	Guangdong Provincial People’s Hospital	INTERVENTIONAL
NCT07368803	Evaluation of the safety, tolerability and efficacy of ineo-Vac-r01, an individualized mRNA therapeutic technology based on tumor neoantigens, for adjuvant treatment in patients with Biliary malignant tumors after radical resection	RECRUITING	Biliary malignant tumors	Yifan Wang	Interventional
NCT07004244	Application of KRAS vaccine in the treatment of KRAS-mutated malignancies	RECRUITING	Malignant tumors	Sichuan University	INTERVENTIONAL
NCT07040943	Clinical trial of IL-22BP safety, tolerability, and antitumor activity in refractory solid tumors.	RECRUITING	Refractory malignant solid tumors|mrna vaccine|interleukin	West China Hospital	INTERVENTIONAL
NCT06195384	Anti-cancer neoantigen mRNA vaccine to treat solid tumors	RECRUITING	Solid tumor, adult	Second affiliated Hospital of guangzhou medical University	INTERVENTIONAL
NCT07092007	NWRD09 for HPV-16 related intraepithelial neoplasia and cervical cancer	RECRUITING	Intraepithelial neoplasia|cervical cancer	Newish technology (Beijing) co., Ltd.	INTERVENTIONAL
NCT06741150	NWRD09 for HPV-16 positive and HPV-16 related intraepithelial neoplasia and cervical cancer patients.	RECRUITING	Intraepithelial neoplasia|cervical cancer	Newish technology (Beijing) co., Ltd.	INTERVENTIONAL
NCT07100210	A clinical trial evaluating IL-22BP/LNP compound in refractory malignant solid tumors for safety, tolerability and activity	RECRUITING	Refractory malignant solid tumors|mRNA vaccine|interleukin	Xingchen Peng	INTERVENTIONAL
NCT06577532	Study of KRAS neoantigen mRNA vaccine (ABO2102) in patients with KRAS -mutated solid tumors	RECRUITING	Pancreatic neoplasms|other solid tumors	Ruijin Hospital	INTERVENTIONAL
NCT06019702	Clinical study of personalized mRNA vaccine encoding neoantigen alone in subjects with advanced digestive system neoplasms	RECRUITING	Digestive system neoplasms	Sir run Run shaw Hospital	INTERVENTIONAL
NCT06026774	Clinical study of personalized mRNA vaccine encoding neoantigen in subjects with resected digestive system neoplasms	RECRUITING	Digestive system neoplasms	Sir run Run shaw Hospital	INTERVENTIONAL
NCT06026800	Clinical study of personalized mRNA vaccine encoding neoantigen in combination with standard first-line treatment in subjects with advanced digestive system neoplasms	RECRUITING	Digestive system neoplasms	Sir run Run shaw Hospital	INTERVENTIONAL
NCT07348042	Study of RGL-270 single drug and combined with Adebelimab in patients in patients at high risk of recurrence after radical treatment of malignant solid tumors	RECRUITING	Tumor, solid	Xian-Jun Yu	INTERVENTIONAL
NCT07306299	A phase I clinical trial of a mRNA vaccine for recurrent or progressive high-grade glioma	RECRUITING	Glioma, high grade	Second affiliated Hospital, school of medicine, Zhejiang University	INTERVENTIONAL
NCT06326736	Study of neoantigen mRNA vaccines in patients with resectable pancreatic cancer	RECRUITING	Pancreatic cancer	Jinling Hospital, China	INTERVENTIONAL
NCT07077356	Application of mRNA vaccine in liver transplantation for hepatocellular carcinoma	RECRUITING	Hepatocellular carcinoma (HCC)	West China Hospital	INTERVENTIONAL
NCT06496373	Clinical study of mRNA vaccine combined with PD-1 inhibitor as adjuvant therapy for postoperative pancreatic cancer	RECRUITING	Pancreatic cancer resectable|chemotherapy-intolerant	Ruijin Hospital	INTERVENTIONAL
NCT06928922	Inhaled mRNA tumor-associated antigen dry Powder vaccine in advanced lung cancer and lung metastasis of solid tumors.	RECRUITING	Advanced lung cancer|lung metastasis	Cancer institute and Hospital, Chinese Academy of medical sciences	INTERVENTIONAL
NCT05579275	Evaluate the safety and tolerability of JCXH-212 monotherapy and combined with toripalimab in the treatment of malignant solid tumors	RECRUITING	Malignant solid tumors	Peking University cancer Hospital and institute	INTERVENTIONAL
NCT05672355	A vaccine booster (GEO-CM04S1) for the prevention of COVID-19 in patients with chronic lymphocytic leukemia	RECRUITING	Chronic lymphocytic leukemia|COVID-19 infection	City of Hope medical center	INTERVENTIONAL
NCT06980155	XP-005 personalized vaccine alone or in combination with toripalimab for the prevention of relapse after remission in acute myeloid leukemia	RECRUITING	Acute myeloid leukemia	Shanghai Jiao tong University school of medicine	INTERVENTIONAL
NCT04534205	A clinical trial investigating the safety, tolerability, and therapeutic effects of BNT113 in combination with pembrolizumab versus pembrolizumab alone for patients with a form of Head and neck cancer positive for human Papilloma virus 16 and expressing the protein PD-L1	RECRUITING	Unresectable Head and neck squamous cell Carcinoma|Metastatic Head and neck Cancer|Recurrent Head and neck cancer	BioNTech SE	INTERVENTIONAL
NCT04163094	Ovarian cancer treatment with a liposome formulated mRNA vaccine in combination with (Neo-)Adjuvant chemotherapy	TERMINATED	Ovarian cancer	University medical center groningen	INTERVENTIONAL
NCT03480152	Messenger RNA (mRNA)-Based, personalized cancer vaccine against neoantigens expressed by the Autologous cancer	TERMINATED	Melanoma|colon cancer|gastrointestinal cancer|genitourinary cancer|hepatocellular cancer	National cancer institute (NCI)	INTERVENTIONAL
NCT05016622	Booster dose trial	TERMINATED	Cancer	Montefiore medical center	INTERVENTIONAL
NCT05359354	Safety and efficacy of personalized neoantigen vaccine in advanced solid tumors	UNKNOWN	Solid tumor	YueJuan Cheng	INTERVENTIONAL
NCT04951323	Impact of the immune system on response to anti-Coronavirus disease 19 (COVID-19) vaccine in Allogeneic stem cell recipients (Covid vaccin allo)	UNKNOWN	Coronavirus disease 2019 (COVID-19)|Hematopoietic neoplasms	University of Liege	INTERVENTIONAL
NCT05192460	Safety and efficacy of personalized neoantigen vaccine in advanced gastric cancer, esophageal cancer and liver cancer	UNKNOWN	Gastric cancer|esophageal cancer|liver cancer	Jianming xu	INTERVENTIONAL
NCT05738447	Application of mRNA immunotherapy technology in hepatitis B virus-related refractory hepatocellular carcinoma	UNKNOWN	Liver cancer|hepatocellular carcinoma	West China Hospital	INTERVENTIONAL
NCT03468244	Clinical study of personalized mRNA vaccine encoding neoantigen in patients with advanced digestive system neoplasms	UNKNOWN	Advanced esophageal squamous carcinoma|gastric adenocarcinoma|pancreatic adenocarcinoma|colorectal adenocarcinoma	Changhai Hospital	INTERVENTIONAL
NCT05761717	Clinical study of mRNA vaccine in patients with liver cancer after operation	UNKNOWN	Posto perative hepatocellular carcinoma	Shanghai Zhongshan Hospital	INTERVENTIONAL
NCT04862806	Safety, efficacy of BNT162b2 mRNA vaccine in CLL	UNKNOWN	Chronic lymphocytic leukemia	Bnai Zion medical center	INTERVENTIONAL
NCT03908671	Clinical study of personalized mRNA vaccine encoding neoantigen in patients with advanced esophageal cancer and non-small cell lung cancer	UNKNOWN	Esophageal cancer|non small cell lung cancer	Stemirna therapeutics	INTERVENTIONAL
NCT05119738	Immune response to third dose of SARS-CoV-2 vaccine in a cohort of cancer patients on active treatment	UNKNOWN	Sars-CoV-2 infection	Pontificia Universidad Catolica de Chile	OBSERVATIONAL
NCT05799612	Phase I study of TH1 dendritic cell immunotherapy for the treatment of cutaneous angiosarcoma	WITHDRAWN	Angiosarcoma	M.D. Anderson cancer center	INTERVENTIONAL

EBV, Epstein–Barr virus; MGMT, O6-methylguanine-DNA, methyltransferase; NSCLC, non-small cell lung cancer; PD-1, programmed death-1; PD-L1, programmed death-ligand 1; HIV, human immunodeficiency virus; HCC, hepatocellular carcinoma; HPV, human papillomavirus; CLL, chronic lymphocytic leukemia; B-NHL, B-cell non-Hodgkin lymphoma; PDAC, pancreatic ductal adenocarcinoma; KRAS, kirsten rat sarcoma viral oncogene homolog.

### 6.2 Tumor-type–specific applications and clinical translation of promising mRNA cancer vaccines

Against this clinical trial landscape, mRNA cancer vaccines have demonstrated distinct application prospects and translation potential across diverse tumor types, with heterogeneous therapeutic performance shaped by inherent tumor immunogenicity, microenvironmental characteristics, and antigen availability. A comparative overview of mRNA cancer vaccines across major tumor types is provided in Table 5.

**TABLE 5:** Comparative overview of mRNA cancer vaccines across different tumor types.

Cancer type	Key biological features	Core rationale	Representative vaccine and clinical stage	Clinical outcomes	Major challenges
Melanoma	High tumor mutational burden (TMB); immunologically “hot” tumor; baseline CD8^+^ T-cell infiltration	Abundant neoantigens; compatible with PD-1 blockade; strong clinical validation	mRNA-4157/V940 + pembrolizumab (phase III); KEYNOTE-942 trial	18-month RFS: 79% (combination) vs. 62% (monotherapy); significantly reduced recurrence risk in adjuvant setting	Antigen selection precision; *in vivo* biodistribution; manufacturing timelines; inter-patient heterogeneity
Pancreatic ductal adenocarcinoma (PDAC)	Immunologically “cold” tumor; dense desmoplastic stroma; poor T-cell infiltration; strong immunosuppressive TME	Personalized neoantigen design; addresses inter-patient mutational heterogeneity; compatible with chemotherapy + ICIs	BNT122 (autogene cevumeran, phase I/II)	Induces durable neoantigen-specific T-cells; prolonged RFS in immune responders; effective in adjuvant/MRD setting	Extremely immunosuppressive TME; low antigen presentation; difficult delivery
Breast cancer	High heterogeneity: most subtypes (e.g., HR+) are immunologically “cold”; TNBC is hard to treat	Multi-antigen encoding capacity; suitable for perioperative/MRD clearance; safety profile confirmed by COVID-19 vaccines	α-Lactalbumin mRNA-LNP; MUC1 mRNA + siMETTL16 (preclinical)	Stable preclinical antitumor activity; suppresses progression/metastasis in TNBC; good safety and tolerability	Limited baseline immunogenicity; need for combinatorial strategies; lack of randomized clinical data
Prostate cancer	Immunologically “cold” tumor; sparse effector T-cell infiltration; immunosuppressive TME	TAA + immune-enhancing module co-delivery; activates ADCC + T-cell cytotoxicity	5T4 + CD70 mRNA-LNP (preclinical)	Triggers synergistic humoral + cellular immunity; enhances CD8^+^ T-cell and NK cell activity; improves CRPC potential	Poor immune priming; single-antigen limitation; requires co-stimulatory molecule integration
Glioblastoma (GBM)	Highly aggressive; high heterogeneity; blood-brain barrier (BBB); immunosuppressive TME	Personalized multi-antigen design; elicits CD4+/CD8+ T-cell responses; virus-associated antigen option	CD133 mRNA-DC vaccine; HCMV-targeted mRNA-DC vaccine (preclinical)	Inhibits glioma stem cells; delays tumor growth; feasible with BBB-penetrating delivery	BBB obstruction; severe immunosuppression; easy immune escape; difficult delivery optimization
Hepatocellular carcinoma (HCC)	Liver tolerogenic microenvironment; chronic liver disease background; LNP naturally targets liver	Spleen-targeted SORT-LNP avoids liver sequestration; encodes immune activators; compatible with ICIs	OX40L mRNA-LNP; spleen-specific mRNA vaccine (preclinical)	Augments CD4+/CD8+ T-cell responses; reverses immunosuppression; avoids liver tolerance	Hepatic immune suppression; off-target hepatocyte uptake; metabolism–immune interference
Colorectal, gastric and other GI cancers	Diverse subtypes; shared TAA expression; modifiable TME	Rapid antigen reconfiguration; enhances DC activation and T-cell recruitment; improves antigen delivery	MAGE-A3 mRNA-LNP (preclinical)	Preclinical tumor inhibition; modulates TME; activates innate + adaptive immunity	Tumor heterogeneity; suboptimal delivery; lack of mature clinical evidence

ADCC, Antibody-Dependent Cellular Cytotoxicity; BBB, blood-brain barrier; CD8+, cluster of differentiation 8 positive; CRPC, castration-resistant prostate cancer; DC, dendritic cell; GBM, glioblastoma multiforme; HCC, hepatocellular carcinoma; HR+, hormone receptor positive; ICIs, immune checkpoint inhibitors; LNP, lipid nanoparticle; MAGE-A3, melanoma-associated antigen 3; MRD, minimal residual disease; OX40L, OX40 ligand; PD-1, programmed death-1; PDAC, pancreatic ductal adenocarcinoma; RFS, recurrence-free survival; siMETTL16, small interfering METTL16; SORT, selective organ targeting; TAA, tumor-associated antigen; TMB, tumor mutational burden; TME, tumor microenvironment; TNBC, triple-negative breast cancer.

#### 6.2.1 Melanoma

Among solid malignancies, melanoma currently presents the most robust evidence for the clinical translation of mRNA cancer vaccines. Mechanistically, the efficacy of LNP-mRNA vaccines in melanoma is underpinned by the tumor’s high mutation burden (TMB), which provides an extensive reservoir of candidate neoantigens. Clinical investigations into personalized neoantigen vaccines have demonstrated that vaccination elicits broad neoantigen-specific T-cell responses; notably, while CD8^+^ T-cell activation is induced, CD4^+^ T-cell responses are particularly pronounced (Ott et al., 2017). The relatively T cell-inflamed microenvironment of melanoma further supports the applicability of mRNA vaccines. Furthermore, the mechanistic complementarity between melanoma pathology and PD-1 blockade has been rigorously validated in clinical settings. Early studies established the durable clinical activity of PD-1 blockade across various solid tumors, including melanoma (Topalian et al., 2012), with the therapeutic value of checkpoint blockade in advanced disease subsequently confirmed (Larkin et al., 2015). Against this backdrop, the rationale for combining mRNA vaccines with PD-1 inhibitors is compelling: the vaccine encodes patient-specific neoantigens which, following translation and presentation *via* MHC-I and MHC-II pathways in antigen-presenting cells, expand polyclonal neoantigen-specific CD8^+^ and CD4^+^ T-cells. Concurrently, PD-1 inhibitors reverse T-cell exhaustion mediated by the PD-1/PD-L1 axis, thereby enhancing the effector function of these vaccine-primed T-cells within the TME (Adhikary et al., 2025; Zoroddu and Bagella, 2025; Gazouli et al., 2025). This concept was further supported by the phase I Lipo-MERIT trial, in which an RNA vaccine induced immune responses in checkpoint-inhibitor-treated patients with melanoma (Sahin et al., 2020). The clinical translation of this mechanistic framework is exemplified by the positive outcomes of mRNA-4157/V940 combined with pembrolizumab in the KEYNOTE-942 trial (Zoroddu and Bagella, 2025; mRNA Vaccine Slows Melanoma Recurrence, 2023). In the randomized, open-label Phase IIb KEYNOTE-942 study, adjuvant treatment with this combination in high-risk resected melanoma significantly reduced the risk of recurrence or death compared to pembrolizumab monotherapy (Zoroddu and Bagella, 2025; mRNA Vaccine Slows Melanoma Recurrence, 2023; Weber et al., 2024). Nevertheless, success in melanoma does not imply the absence of limitations. Personalized mRNA vaccines continue to face challenges regarding the precision of antigen selection, *in vivo* biodistribution of delivery systems, the magnitude and durability of immune responses, manufacturing turnaround times, and inter-patient heterogeneity (Gazouli et al., 2025).

#### 6.2.2 Pancreatic ductal adenocarcinoma

In stark contrast to melanoma, pancreatic ductal adenocarcinoma (PDAC) has historically been regarded as one of the most formidable indications for mRNA vaccines, primarily due to its profoundly immunosuppressive TME. Characterized by a dense desmoplastic stroma, sparse effector T-cell infiltration, poor antigen presentation, and a significant enrichment of immunosuppressive cellular populations, PDAC represents a paradigmatic “immunologically cold” malignancy (Bloom et al., 2025; McMillan and Soares, 2025; Lu Z. et al., 2025). These biological hallmarks explain the generally poor response of PDAC to ICI monotherapy. At present, the most prominent therapeutic candidate in this domain is BNT122 (Hussain and Fareed, 2025). The clinical landmark of this vaccine rests on its pioneering demonstration that personalized mRNA vaccination in human PDAC can elicit detectable neoantigen-specific T-cell responses that directly correlate with clinical outcomes. Recent studies indicate that in the adjuvant setting for resectable PDAC, autogene cevumeran administration drives robust and durable expansion of neoantigen-specific T-cells in a subset of patients. Notably, these immune responders exhibited prolonged recurrence-free survival (RFS), (Nel et al., 2025; Rojas et al., 2023). Extended follow-up at a median of 3.2 years confirmed that vaccine-induced CD8^+^ T cell clones in responders had an estimated average lifespan of 7.7 years, and that responders maintained a significant recurrence-free survival advantage over non-responders (Sethna et al., 2025). These mark a substantial breakthrough in penetrating the immunosuppressive barrier of PDAC. The therapeutic potential of tailored mRNA vaccines in pancreatic cancer is most pronounced among post-operative patients at high risk of recurrence, rather than in those with disseminated disease or heavy tumor burden. This minimal residual disease (MRD) phase offers an optimal window for translating vaccine-induced immunity into tangible clinical benefits (Kang et al., 2023).

#### 6.2.3 Breast cancer

Distinct from melanoma, breast cancer is characterized by profound heterogeneity; the majority of subtypes exhibit limited immunogenicity, with hormone receptor-positive (HR+) variants often manifesting an “immunologically cold” phenotype, which significantly impedes the stability of immunotherapeutic efficacy (Zheng et al., 2026). Integrative analyses of TCGA and METABRIC datasets have pinpointed candidates such as CD74, IRF1, and PSME2, which exhibit overexpression, amplification, or mutation in breast cancer and correlate with patient prognosis and immune infiltration, suggesting substantial vaccine development potential (Li R. Q. et al., 2022). Immunoinformatics approaches applied to CA-125-related mutations have successfully identified potentially immunogenic CD8^+^ T-cell epitopes, leading to the construction of self-adjuvanting mRNA vaccines incorporating CD40L and MHC-I targeting domains to enhance dendritic cell (DC) cross-presentation (Lu et al., 2023). Additionally, TAAs such as VEGFR2 and c-MET have been selected due to their overexpression; epitope screening for dual T-cell and B-cell responses, validated by molecular docking and dynamic simulations, confirmed strong MHC interaction and complex stability (Ghayoumian et al., 2025). For triple-negative breast cancer (TNBC), a subtype presenting significant therapeutic challenges, strategies combining METTL16-targeting nano-formulations with mRNA vaccines have been explored (Wang et al., 2025). An α-lactalbumin mRNA-LNP vaccine developed for TNBC induced specific IgG antibodies and IFN-γ-secreting T-cell responses in murine models, inhibiting 4T1 tumor growth under prophylactic conditions (He et al., 2024). Furthermore, combining this vaccine with surgical resection effectively suppressed progression and metastasis (He et al., 2024), suggesting that mRNA vaccines may possess unique value in the perioperative setting and for clearing minimal residual disease (MRD), rather than solely for advanced unresectable cases. Another study demonstrated that combining a METTL16-downregulating nano-formulation (LNP/siMETTL16) with a MUC1-encoding mRNA vaccine (LNP/mMUC1) significantly inhibited subcutaneous tumor growth by 66.0% in a TNBC model (Wang et al., 2025). Moreover, organ-selective targeting (SORT-LNP/siMETTL16) combined with vaccination markedly reduced lung metastasis (Wang et al., 2025). In terms of delivery optimization, macroporous hydrogel-based platforms have been developed to recruit and modulate DCs *in situ*, thereby enhancing cytotoxic T-lymphocyte (CTL) responses and demonstrating anti-tumor activity in 4T1 models (Zhou J. et al., 2025).

#### 6.2.4 Prostate cancer

Prostate cancer is characteristically classified as an immunologically “cold” malignancy, defined by a TME exhibiting sparse effector T-cell infiltration and sustained immunosuppressive signaling. Consequently, clinical responses to monotherapies, such as ICIs, remain generally suboptimal (Xu et al., 2024). Against this backdrop, mRNA vaccines offer a distinct immunotherapeutic intervention compared to conventional ICIs. While Sipuleucel-T stands as the sole regulatory-approved therapeutic vaccine for prostate cancer, its complex manufacturing process and restricted clinical applicability underscore an urgent unmet need for novel, scalable, and standardized vaccine platforms. Exemplifying this combinatorial strategy is the 5T4 and CD70 mRNA-LNP vaccine. 5T4, a TAA highly expressed in various malignancies yet minimally present in normal tissues, offers superior tumor selectivity, rendering it an ideal target for inducing specific immune recognition (Cao et al., 2025). Consequently, within mRNA vaccine architecture, CD70 acts not merely as a “supplementary antigen” but as a functional immune-enhancing module (Cao et al., 2025). In murine prostate cancer models, co-encapsulation of 5T4 and CD70 mRNA within LNPs elicited superior humoral and cellular immune responses compared to antigen-only strategies, translating into significantly enhanced tumor suppression and survival benefits (Cao et al., 2025).

#### 6.2.5 Glioblastoma

Glioblastoma (GBM) is a highly aggressive primary brain malignancy characterized by frequent recurrence and exceptionally poor prognosis despite standard-of-care interventions (Barati et al., 2025). However, clinical translation faces formidable hurdles. First, the blood-brain barrier (BBB) severely restricts the effective delivery of systemically administered mRNA and its vectors into the central nervous system (CNS). Second, the local immune microenvironment of GBM is profoundly immunosuppressive, dominated by tumor-associated macrophages/microglia, which inhibit effector T-cell infiltration and functional persistence. Third, significant inter-patient and intratumoral heterogeneity render single-antigen strategies highly susceptible to immune escape (Strika et al., 2024; Karimi-Sani et al., 2024; Li H. et al., 2025). Despite these barriers, a phase Ib clinical study demonstrated that personalized neoantigen vaccines in newly diagnosed GBM can elicit polyfunctional neoantigen-specific CD4^+^ and CD8^+^ T cell responses that infiltrate the intracranial tumor, establishing early proof-of-concept that vaccines can favorably alter the immune milieu of this disease (Keskin et al., 2019). Transcriptomic and immune subtype analyses have successfully identified potential tumor antigens suitable for mRNA vaccine development, further defining immune subtypes more likely to derive clinical benefit (Zhong et al., 2021). Subsequent studies have validated the feasibility of GBM/glioma mRNA vaccine development from both tumor-antigen and immunophenotyping perspectives, supporting the concurrent advancement of antigen screening and patient stratification (Chen et al., 2022). Dendritic cell (DC) vaccines loaded with CD133 mRNA induced CD4^+^ and CD8^+^ T-cell activation in humanized GBM murine models, inhibiting glioma stem cell proliferation and tumor growth (Do et al., 2020). Another study used immunopeptidomics to identify MHC-I-associated peptide antigens presented during tumor-macrophage co-evolution; mRNA vaccines based on these targets significantly delayed GBM growth *in vivo*, leading to tumor clearance in some models (Cui et al., 2025). Regarding delivery system optimization, sequential selective organ-to-cell targeting strategies for glioma mRNA vaccines have been developed. These emphasize engineering delivery vehicles to enhance mRNA accumulation in target tissues and cells, offering novel technical avenues to bypass the BBB and improve targeted immune cell delivery (Shi et al., 2025). Studies suggest that combining vaccines with ICIs, TGF-β pathway modulation, or other immunomodulatory agents may yield stronger synergistic effects (Shi et al., 2025; Zhu et al., 2023; Tu et al., 2023). In murine glioma models, the addition of TGF-β2 inhibitory oligonucleotides as molecular adjuvants enhanced anti-glioma immune responses (Tu et al., 2023). Other research indicates that targeting IDH1-related strategies combined with anti-PD-1 therapy can further improve outcomes (Shi et al., 2025). Human cytomegalovirus (HCMV)-related antigens may offer novel therapeutic targets for a subset of GBM patients (Söderberg-Naucler et al., 2025). Early studies of mRNA-DC vaccines targeting HCMV antigens such as pp65 have shown signals of survival benefit (Söderberg-Naucler et al., 2025).

#### 6.2.6 Hepatocellular carcinoma

Unlike other solid tumors, the idiosyncrasy of hepatocellular carcinoma (HCC) primarily stems from the liver’s unique immunological niche. The hepatic microenvironment is inherently tolerogenic; constant exposure to gut-derived antigens *via* the portal circulation skews the local immunological balance toward suppression. Furthermore, HCC often arises against a backdrop of chronic hepatitis, cirrhosis, or metabolic liver disease, thereby compounding the complexity of both the tumor immune microenvironment (TIME) and systemic immune status (Fu et al., 2023; Wang et al., 2024). Additionally, the natural tropism of LNPs for the liver presents a pronounced “double-edged sword” in HCC: while it facilitates drug accumulation in the organ, it simultaneously risks excessive uptake by non-malignant hepatocytes and liver-resident immune cells. This off-target sequestration can compromise the efficiency of antigen delivery and subsequent immune activation (Li H. et al., 2025; Elliott et al., 2025).

Currently, several mRNA vaccine candidates for HCC have progressed to experimental validation. For instance, an LNP-formulated, optimized OX40L mRNA vaccine demonstrated the capacity to augment CD4^+^ and CD8^+^ T-cell responses within the TME of murine HCC models, thereby inhibiting tumor progression and prolonging survival (Deng et al., 2022). These findings suggest that utilizing mRNA to encode immune-activating molecules can not only bolster local T-cell responses but also partially reverse the immunosuppressive state. In a divergent approach, recent advances in selective organ targeting (SORT) have enabled the design of spleen-specific LNPs that actively bypass liver accumulation. Studies have shown that by incorporating specific internal charge adjustments, mRNA vaccines can be precisely redirected to splenic dendritic cells rather than hepatocytes (Cheng et al., 2020). This strategy allows for robust antigen presentation in peripheral immune organs, thereby avoiding the weakening effects of the liver’s natural tolerance environment and eliciting potent systemic anti-tumor immunity.

Regarding target discovery, bioinformatic analyses have identified multiple classes of potential mRNA vaccine antigens for HCC. Candidates such as PES1, MCM3, PPM1G, and KPNA2 have been identified. These molecules are linked to both antigen-presenting cell infiltration and poor prognosis, highlighting their potential as vaccine targets. (Fu et al., 2023). By further integrating potential tumor targets with immunophenotyping, researchers have identified candidate genes, including CDC20, CDK1, DLGAP5, MELK, NCAPG, NUSAP1, and TOP2A, and postulate that distinct immunophenotypes may necessitate divergent vaccine strategies (Wang et al., 2024). In addition, recent studies have proposed that improving the liver microenvironment for HCC by modulating bile acid metabolism and increasing ursodeoxycholic acid levels may enhance the efficacy of mRNA vaccines. These strategies suggest that combination therapy for HCC may need to go beyond classical ICIs and incorporate the unique dimension of liver metabolism-immune interactions (Llovet et al., 2022).

#### 6.2.7 Colorectal, gastric, and other gastrointestinal cancers

Beyond the aforementioned malignancies, colorectal cancer (CRC), gastric cancer (GC), and other gastrointestinal (GI) tumors have demonstrated favorable compatibility with mRNA vaccine platforms, though robust clinical translation evidence remains broadly absent. In CRC, researchers have developed novel LNPs based on DMKD and phosphatidylserine to deliver MAGE-A3-encoding mRNA. Significant reductions in both tumor volume and weight were observed in preclinical models, indicating that vector optimization can substantially enhance tumor antigen delivery and corresponding anti-tumor immune responses (Choi et al., 2024). GI tumors as a whole are considered suitable candidates for mRNA vaccinology, primarily because the platform concurrently triggers both innate and adaptive immunity, while allowing for the rapid reconfiguration of encoded antigens tailored to diverse tumor types (Zhang A. et al., 2023). The therapeutic value of mRNA vaccines in GI cancers extends beyond their role as mere antigen vectors; they actively modulate the TME by influencing antigen presentation, driving DC activation, and facilitating T-cell recruitment (Zhang A. et al., 2023).

Viewed from a broader perspective in solid tumor oncology, therapeutic mRNA vaccines have shown the potential to promote antigen-specific T-cell expansion and tumor infiltration in various cancers; however, clinical benefits differ greatly among tumor types (Elliott et al., 2025). Studies on virus-mimicking mRNA vaccines have shown a remarkable ability to enhance the proliferation and infiltration of antigen-specific CD8^+^ T-cells within tumors in model systems. This suggests that combining smart delivery methods with innate immune activation strategies can greatly increase the anti-tumor effectiveness of mRNA vaccines (Meng et al., 2021). Furthermore, improvements in mRNA structure, such as nucleoside modifications and codon optimization, along with advances in delivery systems like LNPs, have been shown to significantly enhance stability, translational efficiency, and immunogenicity. (Mei and Wang, 2023; Ni, 2023). Nonetheless, the clinical application of cancer mRNA vaccines is currently hindered by ubiquitous challenges, including tumor heterogeneity, TME immunosuppression, suboptimal delivery efficacy, and difficulties in accurately identifying true beneficiary populations (Li H. et al., 2025). As these specific hurdles persist in GI malignancies like CRC and GC, their development, despite having a solid platform foundation, remains far from mature clinical deployment.

## 7 Challenges and future perspectives of mRNA cancer vaccines

### 7.1 Challenges in delivery systems and directions for optimization

mRNA vaccines hold substantial promise for cancer treatment; however, their clinical translation remains limited by insufficient delivery efficiency and poor *in vivo* stability (Al Fayez et al., 2023). Owing to their large molecular size and negative charge, mRNA molecules cannot readily cross the cell membrane by passive diffusion, which restricts intracellular uptake, compromises stability, and reduces protein expression efficiency (Wang et al., 2022). In addition, as single-stranded nucleic acids, mRNA transcripts are highly susceptible to rapid degradation by ribonucleases (RNases), resulting in a short circulation half-life and inadequate delivery to target cells (Han et al., 2023). Naked mRNA is also intrinsically immunogenic and may induce excessive innate immune activation, thereby interfering with translation of the encoded antigen (Gao et al., 2021). Therefore, a central issue in the development of mRNA cancer vaccines is how to preserve mRNA integrity while improving membrane translocation, cytosolic release, and target-specific delivery.

To address the intrinsic instability, degradability, and limited translational efficiency of mRNA, molecular engineering has become an important optimization strategy. Incorporation of modified nucleosides such as pseudouridine (ψ) and N1-methylpseudouridine (m1ψ) can reduce recognition by pattern-recognition receptors, including TLR7/8, thereby attenuating innate immune activation while enhancing mRNA stability and translational efficiency (To and Cho, 2021; Li et al., 2022b). Likewise, optimization of the 5′ cap structure, 3′ poly(A) tail, untranslated regions, and codon usage helps protect mRNA from exonuclease-mediated degradation, improves ribosome recruitment, and increases protein output (Mohamad Razif et al., 2023).

Beyond chemical modification of the mRNA molecule itself, the development of efficient and safe delivery systems represents another indispensable pillar for the successful application of mRNA in cancer immunotherapy (Prinindya et al., 2026). An ideal carrier must shield mRNA from enzymatic degradation, facilitate its traversal across the plasma membrane, and promote endosomal escape into the cytoplasm for translation (Neill et al., 2024). Among existing delivery platforms, LNPs remain the most extensively investigated and clinically mature system for mRNA delivery (Lin et al., 2024; Jacob et al., 2024). A typical LNP comprises ionizable lipids, phospholipids, cholesterol, and polyethylene glycol (PEG)-conjugated lipids, which self-assemble into nanoparticles through microfluidic mixing to encapsulate mRNA with high efficiency (Eygeris et al., 2022). Within this architecture, ionizable lipids undergo protonation in the acidic endosomal environment and interact with the endosomal membrane, facilitating mRNA release into the cytosol—a process termed “endosomal escape” that is widely regarded as the rate-limiting step governing delivery efficacy (Paroor et al., 2025). LNPs have achieved remarkable success in COVID-19 vaccination and have since been rapidly adapted for cancer vaccine research (Uchida, 2026).

Nevertheless, significant limitations persist in the oncological setting. Following systemic administration, LNPs predominantly accumulate in the liver, which not only restricts targeted delivery to immune organs such as the spleen and lymph nodes but also raises concerns regarding hepatotoxicity (Loughrey and Dahlman, 2022). Consequently, a central research priority lies in the rational design and optimization of delivery systems to overcome mRNA instability, achieve efficient *in vivo* delivery, and precisely modulate immune activation.

To this end, ongoing efforts are directed toward rationally engineering LNP composition and structural parameters to improve biodistribution, cellular uptake, and cytoplasmic release, while striking an appropriate balance between immunostimulation and tolerability (Uchida, 2026). One innovative approach involves co-assembling STING-activating polymers (PD) with lipid materials and mRNA into lipid-like nanoparticles (PD LNPs), which not only enhance lymphatic delivery of mRNA but also potentiate immune activation through the STING pathway while maintaining favorable tolerability (Zhang M. et al., 2025). An alternative strategy entails co-loading the cGAS agonist Svg3 with antigen-encoding mRNA within LNPs, leveraging type I interferon responses to augment antigen presentation and CD8^+^ T cell responses; this combination has demonstrated enhanced antitumor efficacy when administered alongside ICIs (Zhou S. et al., 2025). These findings underscore that the optimization of delivery systems extends well beyond mRNA protection, increasingly serving to direct the mode and magnitude of immune activation, with the overarching aim of maximizing therapeutic benefit while minimizing off-target effects and systemic toxicity.

In addition to LNP refinement, novel delivery carriers are under active development to simultaneously improve delivery efficiency, mitigate inflammatory side effects, and enhance targeting precision. Polymeric nanoparticles, lipid–polymer hybrid systems, and coacervate-based delivery platforms have all been explored as alternative mRNA delivery modalities (Yang et al., 2023; Forenzo and Larsen, 2023). For instance, polymeric nanoparticles (PNPs) constructed from alternating copolymers exhibit negligible inflammatory side effects *in vivo* while effectively delivering mRNA cancer vaccines and eliciting robust CD8^+^ T cell-mediated antitumor immunity (Huang et al., 2023). Meanwhile, fluoroalkyl-grafted polyethyleneimine (F-PEI) has been synthesized for mRNA delivery; this polymer not only facilitates intracellular mRNA transport but also activates the TLR4 signaling pathway, enabling dendritic cell maturation and antigen presentation without the need for exogenous adjuvants (Li Y. et al., 2025). Moreover, fine-tuning nanoparticle size, surface charge, and internal architecture can profoundly influence biodistribution and cellular uptake kinetics. Specifically, controlling mRNA-LNP dimensions within the 200–500 nm range has been shown to more effectively target splenic dendritic cells, thereby amplifying immune responses (Sasaki et al., 2022). Taken together, the future trajectory of delivery system development is no longer confined to merely improving encapsulation efficiency; rather, it is oriented toward the systematic optimization of stable, controllable, and highly efficient intracellular release and expression of mRNA in target cells, thereby providing a more reliable technological foundation for the clinical application of mRNA cancer vaccines.

### 7.2 Challenges in efficacy and clinical translation and pathways to breakthrough

Although mRNA cancer vaccines have demonstrated favorable immunogenicity in early-stage investigations, a central contradiction persists: the disconnect between immune responses and clinical benefit. Numerous early-phase clinical trials have confirmed that mRNA vaccines can elicit antigen-specific T cell responses or humoral immunity; however, these immunological endpoints do not invariably translate into durable improvements in relapse-free survival (RFS) or overall survival (OS) (Katopodi et al., 2024; Vlachostergios, 2025). Prior systematic reviews have noted that while mRNA cancer vaccines are capable of generating detectable immune responses across multiple malignancies, overall clinical efficacy remains limited, suggesting a considerable gap between “inducing an immune response” and “achieving definitive clinical benefit” (Heine et al., 2021). Representative recent studies have further illustrated this point: even when personalized mRNA neoantigen vaccines successfully induced neoantigen-specific T cell responses in pancreatic cancer patients, long-term and stable clinical benefit still awaits validation through larger cohorts and extended follow-up (Rojas et al., 2023). These observations indicate that the critical bottleneck in mRNA cancer vaccine development has shifted from demonstrating immunological activity to ensuring that such activity reliably translates into meaningful clinical outcomes.

The foremost contributor to this disconnect is the persistent attenuation of vaccine-induced immunity by the immunosuppressive tumor microenvironment (TME) together with tumor-intrinsic and adaptive immune evasion mechanisms. Even when vaccines successfully prime tumor-specific T cells, effector cells infiltrating the tumor may be functionally restrained by the suppressive milieu, rendering them unable to exert full cytotoxic activity. Interactions between tumor cells and other components of the microenvironment further complicate this picture; for example, hybrid cells formed through the fusion of tumor cells and M2-polarized macrophages have been shown to acquire enhanced migratory capacity and tumorigenicity while exhibiting heterogeneous resistance to antigen-specific T cell killing, thereby facilitating immune escape (Minowa et al., 2023). Additionally, metabolic reprogramming within tumors, such as altered lipid metabolism, not only sustains tumor growth but also modulates the behavior of infiltrating immune cells, fostering immunosuppression and evasion (Pascual and Benitah, 2024). Compounding these challenges, substantial intratumoral heterogeneity is closely linked to both immune activity and immune escape (Lapuente-Santana et al., 2024) and represents a core obstacle to immunotherapy success. Antigen expression varies across distinct tumor cell subpopulations, and single-antigen strategies are particularly vulnerable to selective immune evasion during treatment. It has been emphasized that cold or immunologically compromised tumors frequently exhibit insufficient immune infiltration coupled with amplified immunosuppressive signaling, rendering single-modality immunostimulation insufficient for durable therapeutic benefit (Galon and Bruni, 2019). Consequently, optimization strategies for mRNA cancer vaccines have progressively evolved toward integrated approaches that simultaneously activate antitumor immunity, relieve immunosuppression, and curtail immune escape. In this context, designing multi-target, multi-antigen vaccines is particularly important, as conventional single-target vaccines are prone to failure due to antigen loss, mutational drift, or subclonal expression patterns. The architecture of neoantigens, including their concurrent expression and clonality, determines the immunogenicity of individual neoantigens and drives immune evasion in tumors with heterogeneous neoantigen expression. Therefore, designing vaccines informed by a comprehensive understanding of the tumor neoantigen landscape has emerged as a promising paradigm. In a lung cancer model, for instance, RNA-based therapeutic vaccination targeting immunosuppressive T cell responses acted synergistically with ICIs to control tumors harboring subclonal neoantigen expression (Roerden et al., 2024). Consistent with this, integrating next-generation sequencing with computational epitope prediction enables the identification and prioritization of patient-specific neoantigens, which can then be delivered *via* mRNA platforms to elicit broad and synergistic T cell responses (Prabhu et al., 2026). Such multi-antigen vaccines are designed to simultaneously target both clonal and subclonal antigens present within the tumor, thereby narrowing the window for immune evasion arising from intratumoral heterogeneity. Furthermore, combining mRNA vaccines with agents that target the immunosuppressive microenvironment can additionally enhance vaccine potency. Incorporation of PD-L1 small interfering RNA (siRNA) into cationic liposome-encapsulated tumor mRNA vaccines has been demonstrated to downregulate PD-L1 expression on both dendritic cells and tumor cells, thereby synergistically augmenting CD8^+^ T cell cytotoxicity and preventing tumor immune escape (Zhou et al., 2025c). Accordingly, future mRNA vaccine design should thoroughly account for tumor heterogeneity by adopting multi-target strategies in conjunction with adjunctive approaches capable of remodeling the immunosuppressive microenvironment, thereby maximizing durable and effective antitumor immune responses. Concurrently, modifying the route of administration, for example, *via* subcutaneous, intradermal, intratumoral, or intrasplenic injection, may further modulate the intensity and quality of immune responses, offering supplementary strategies to counteract TME-mediated suppression (Liu et al., 2023).

A second major barrier to clinical translation is insufficient patient stratification, which is closely linked to the limited accuracy of current neoantigen prediction frameworks. The theoretical foundation of personalized neoantigen vaccines rests on the ability to identify, from a vast pool of tumor mutations, those that are genuinely immunogenic. However, an enormous attrition rate exists between genomic mutation detection and the generation of truly immunogenic neoantigens. Although high-throughput sequencing and bioinformatic prediction have markedly broadened the candidate neoantigen repertoire, only a small fraction of predicted targets are capable of inducing effective T cell responses and translating into therapeutic benefit (Blass and Ott, 2021). This difficulty stems largely from the reliance of current prediction algorithms on MHC binding affinity, whereas high binding affinity does not equate to high immunogenicity. Systematic investigations have further demonstrated that the immunogenicity of tumor epitopes is governed not solely by MHC binding capacity but also by antigen processing efficiency, presentation dynamics, and T cell receptor recognition, rendering single-metric binding scores insufficient for accurately identifying efficacious neoantigens (Wells et al., 2020). This challenge is particularly pronounced in tumors with low mutational burden. In non–T-cell-inflamed tumors such as pancreatic cancer, the limited number of available mutations frequently results in an insufficient pool of high-quality candidate neoantigens, even when personalized approaches are employed (Rojas et al., 2023). Therefore, one of the keys to improving therapeutic outcomes lies in refining neoantigen prediction algorithms and more tightly coupling bioinformatic screening with *in vitro* functional validation to enhance the quality of neoantigens entering the vaccine design pipeline. Meanwhile, emerging strategies such as immunopeptidomics, multi-omics integration, and deep learning models incorporating antigen processing and T-cell recognition features may help improve candidate prioritization and, in turn, support more precise patient stratification (Ott et al., 2017; Wells et al., 2020).

A third, more practical barrier is the manufacturing and implementation burden associated with personalized mRNA vaccines. Individualized neoantigen vaccines typically require a complex, multi-step workflow encompassing tumor biopsy, sequencing, bioinformatic analysis, neoantigen selection, mRNA synthesis and formulation, and quality control, which is an intrinsically time-consuming process. Neoantigen vaccine development depends not only on accurate antigen identification but is also jointly constrained by manufacturing cycle times, production complexity, and the logistical challenges of individualized implementation (Schumacher et al., 2019). For patients with rapidly progressing advanced disease, protracted manufacturing timelines may cause them to miss the optimal therapeutic window. Simultaneously, the reliance of individualized workflows on high-throughput sequencing, bioinformatic analysis, and small-batch GMP production renders the overall process prohibitively expensive, restricting broad accessibility and the feasibility of insurance reimbursement (Blass and Ott, 2021). These logistical constraints also influence which clinical settings are most suitable for personalized vaccine deployment. Personalized vaccines appear more feasible in cases with lower tumor burden and sufficient treatment time, such as postoperative adjuvant settings or disease states at high risk of recurrence, where the therapeutic window is broader, and the probability of completing individualized production is higher (Rojas et al., 2023). At the same time, improving manufacturing automation, platform standardization, and process harmonization to reduce production times and costs is essential for expanding clinical use accessibility (Magoola and Niazi, 2025).

Beyond these primary barriers, institutional challenges in clinical trial design and regulatory translation further complicate late-stage development. The majority of current early-phase trials focus primarily on safety and immunogenicity, while the question of how to effectively correlate immunological endpoints, such as T cell proliferation and cytokine secretion, with clinical outcomes (RFS and OS), and how to establish biomarkers predictive of therapeutic response, remains pivotal for advancing these vaccines into standard-of-care regimens (Katopodi et al., 2024; Vlachostergios, 2025). This challenge is especially acute in studies combining mRNA vaccines with PD-1/PD-L1 inhibitors, where the design of appropriate control arms, determination of optimal dosing sequences and schedules, and rigorous assessment of genuine synergistic effects all demand more sophisticated trial architectures (Yang et al., 2025). In addition, personalized neoantigen vaccines are highly customized from candidate antigen identification through GMP manufacturing and quality control, which creates new challenges for traditional statistical frameworks in clinical trials and for established regulatory pathways (Magoola and Niazi, 2025). Unlike conventional off-the-shelf biologics, these products require GMP-compliant CMC control across highly individualized, small-batch production runs, which complicates standardization, lot release, and turnaround time. Key quality attributes, including RNA integrity, residual dsRNA, and LNP formulation consistency, must be tightly controlled to ensure reproducibility and safety. Emerging solutions such as modular platform-based manufacturing, in which only the antigen-encoding sequence is changed while the core process remains standardized, may improve scalability and regulatory comparability, while decentralized or automated production systems could help shorten delivery timelines for patient-specific vaccines (Jiang and Lu, 2025). Consequently, the definitive clinical breakthrough of mRNA cancer vaccines will depend not only on the immunological design of the vaccine itself but also on more accurate neoantigen selection, more judicious identification of clinical scenarios, more efficient personalized manufacturing platforms, and the establishment of compatible clinical evaluation and regulatory frameworks.

### 7.3 Immunogenicity regulation and safety considerations

The successful clinical use of mRNA vaccines in cancer therapy relies not only on their ability to generate strong antitumor immune responses but also on the precise regulation of immunogenicity and careful safety measures. mRNA molecules possess natural immunostimulatory properties that activate the innate immune system *via* pattern recognition receptors, such as TLRs and RIG-I-like receptors. While this feature initiates adaptive immunity, excessive activation can trigger significant inflammatory responses, systemic side effects, and reduced mRNA translation efficiency, ultimately reducing vaccine effectiveness. Therefore, balancing immune activation and inflammation is a key challenge in mRNA vaccine development (Weidensee and Sahu, 2025).

Nucleoside modification and molecular structure optimization form the core strategies for reducing nonspecific innate immune activation and increasing expression efficiency. Replacing uridine with pseudouridine (Ψ) significantly lowers mRNA immunogenicity and reduces type I interferon production, thus enhancing stability and translational output while preventing excessive inflammatory responses responses (Mei and Wang, 2023; Li et al., 2022b). Optimizing untranslated regions and codon usage further enhances translational efficiency and improves safety profiles. However, for cancer vaccines, eliminating immunostimulatory potential may reduce therapeutic effectiveness. Current approaches, therefore, focus on co-delivering modified, low-immunogenicity mRNA with carefully engineered adjuvants to enable controlled yet strong immune activation. (Jiang et al., 2024). Within this framework, delivery vehicles have evolved beyond their traditional role as passive mRNA protectants to become active participants in immunogenicity regulation. LNPs themselves exert adjuvant-like effects by promoting antigen-presenting cell maturation and antigen presentation (Weidensee and Sahu, 2025). Beyond conventional LNPs, novel polymeric nanoparticles have shown promise in maintaining effective immunostimulation while reducing inflammatory side effects (Huang et al., 2023). In cancer patients, achieving this equilibrium is particularly critical, as disease-related or treatment-induced immunosuppression may both dampen vaccine responsiveness and heighten susceptibility to inflammatory complications (Ligumsky et al., 2022).

Despite the mRNA platform’s generally favorable modulability, clinical use requires careful attention to potential safety risks. mRNA-LNP vaccines raise concerns, including allergic or immune reactions caused by lipid components like PEG, possible toxicity of the translated protein products, and systemic inflammation resulting from non-targeted effects delivery (Szebeni, 2025). The heterogeneous immune status of cancer patients further complicates safety assessment. Multiple studies have demonstrated that mRNA-based COVID-19 vaccines are generally well tolerated in oncology populations. One study enrolling 326 solid tumor patients receiving active anticancer treatment reported good tolerability of BNT162b2 with no serious adverse events, although antibody titers in patients undergoing chemotherapy were significantly lower than those in healthy controls, and seronegative rates were higher (Ligumsky et al., 2022). Another investigation involving 284 cancer patients showed that adverse reactions following a third dose of mRNA-1273 were predominantly mild and transient, with serious treatment-related events within 28 days being exceedingly rare (<2%); however, marked intertumortype differences in immune response were observed, with patients bearing lymphoid malignancies exhibiting weaker responses than those with solid tumors (Giuliano et al., 2023). Similar studies have further suggested that vaccine responsiveness in cancer patients is closely associated with prior treatment exposure, tumor type, and immune status, which is an important reason why safety evaluation in the oncology setting is more complex than in general prophylactic vaccination (Hu et al., 2021). Safety concerns should therefore not be narrowly construed as short-term adverse event rates. Still, they should encompass the controllability of immune responses across diverse patient populations, the accumulation of long-term risks, and the stability of the therapeutic window (Szebeni, 2025). Beyond short-term adverse events, the long-term safety of mRNA cancer vaccines also warrants attention, particularly regarding immune tolerance and potential autoimmunity. Repeated or prolonged antigen exposure may attenuate antitumor efficacy by promoting T-cell dysfunction or peripheral tolerance, a risk that may require particular attention in next-generation RNA platforms with extended antigen expression (Wherry, 2011). Vaccines targeting shared tumor-associated antigens may also theoretically increase the risk of breaking self-tolerance, whereas this concern is expected to be lower for personalized neoantigen vaccines (Finn, 2008; Butterfield, 2015). Moreover, durable therapeutic benefit depends not only on acute effector responses but also on the generation of long-lived memory T cells (Pardi et al., 2018). Current evidence suggests that memory T-cell persistence is shaped by antigen exposure dynamics, co-stimulatory signaling, and cytokine support such as IL-7 and IL-15 (Kaech and Cui, 2012; Jameson and Masopust, 2018). These observations highlight the importance of achieving balanced, rather than excessive, immune activation in mRNA cancer vaccine design.

Looking ahead, safety optimization of mRNA cancer vaccines should advance concurrently across materials, delivery, and clinical monitoring dimensions. On one front, the development of safer biodegradable lipids and non-PEGylated polymers is needed to mitigate allergic and systemic toxicity risks (Jiang and Lu, 2025; Wu Y. et al., 2025). On the other hand, organ-targeted delivery approaches, such as enhancing directed transport to immune organs, including the spleen, can reduce non-target tissue exposure, thereby improving efficacy while lowering systemic side effects (Jiang and Lu, 2025; Wu Y. et al., 2025). Moreover, sustained surveillance of long-term risks, including cardiovascular adverse events, inflammatory sequelae, and IgG4 accumulation, warrants strengthening. Overall, immunogenicity regulation and safety management should not be viewed as impediments to the advancement of mRNA cancer vaccines but rather as essential prerequisites for their evolution into controllable, long-term therapeutic modalities.

### 7.4 Future research directions

#### 7.4.1 Vaccine platforms integrating gene editing and nanotechnology

The convergence of gene editing and nanodelivery platforms advances mRNA vaccines by improving precision and potency. Traditional mRNA vaccines encode tumor antigens to activate immunity. Adding gene-editing tools like CRISPR-Cas9 allows direct, precise genome modulation of tumor or immune cells, overcoming the immunosuppressive TME. Future integration of CRISPR-Cas9 could boost the antitumor effects of mRNA vaccines (Yaremenko et al., 2025). This paradigm transcends mere antigen presentation, providing novel tools to reshape the tumor immune landscape. LNP play a pivotal role in mRNA vaccine delivery, particularly within the domain of precision oncology (Jacob et al., 2024). Future studies should develop intelligent nanoplatforms that efficiently co-deliver multiple functional mRNA species, like antigen-encoding and gene-editing mRNA, maintaining stability and targeting *in vivo* for more potent next-generation vaccines platforms. (Pan et al., 2024).

#### 7.4.2 Development of novel delivery carriers and adjuvant technologies

Developing efficient, safe, targeted delivery carriers is crucial for mRNA cancer vaccines. Future efforts will expand beyond traditional LNPs to include polymeric nanoparticles, peptide carriers, virus-like particles, and exosomes. (Miao et al., 2021). One study developed a virus-mimicking mRNA vaccine platform with a phospholipid bilayer encasing mRNA, CpG oligonucleotides, and positively charged proteins. It facilitated efficient mRNA delivery and used CpG as a potent adjuvant with checkpoint-blocking activity, showcasing the carrier’s multifunctional design potential (Meng et al., 2021). Optimizing auxiliary technologies like adjuvants is essential. Utilizing carrier materials’ natural immunostimulatory properties, such as specific cationic lipids or polymers, to serve as self-adjuvants provides a promising way to simplify vaccine formulations and boost immunity activation (Zhang and Xia, 2021). Through engineering modifications, carriers may also be designed to specifically target dendritic cells or lymph nodes, thereby greatly improving antigen presentation efficiency and reducing off-target effects, making this an important focus of future delivery system design (Kirtane and Traverso, 2025).

#### 7.4.3 Exploration of multimodal combination immunotherapy strategies

Given the complexity of tumor immune escape mechanisms, multimodal combination strategies integrating mRNA vaccines with other immunotherapies and conventional treatments will likely become an inevitable direction in future clinical research and a key route toward improved efficacy. A large body of preclinical and clinical evidence indicates that mRNA vaccines may act synergistically with ICIs (Miao et al., 2021). Vaccines can activate and expand tumor-specific T cells, whereas checkpoint inhibitors release these T cells from functional suppression, thereby generating stronger and more durable antitumor immunity (Liu et al., 2024). Beyond checkpoint inhibitors, combinations of mRNA vaccines with adoptive cell therapies such as CAR-T, cytokine therapy, chemotherapy, radiotherapy, and targeted treatment are also being actively investigated (Pati et al., 2025). Future studies should use biomarkers to identify patient populations most likely to benefit, thereby facilitating the clinical application of mRNA vaccines (Lan et al., 2025). Through such combination strategies, the ultimate goal is to enhance the efficacy of mRNA cancer vaccines and overcome tumor heterogeneity and immunosuppression (Liu et al., 2023).

## 8 Conclusion

Overall, mRNA cancer vaccines are transforming cancer immunotherapy by providing a flexible, personalized platform. Unlike traditional methods, they enable rapid production of tumor-specific and immunomodulatory antigens, with improvements in sequence design and delivery that fine-tune antigen expression and immune responses. Their antitumor effects depend not only on antigen recognition but also on the delivery system’s support for cellular uptake, innate sensing, and antigen cross-presentation, which influence T-cell activation, tumor infiltration, and the conversion of the immunosuppressive TME into a T-cell-inflamed environment. This interplay among antigen design, immune regulation, and TME modification sustains their therapeutic potential. Despite encouraging results, major challenges hinder the clinical success of mRNA cancer vaccines. Precise, timely antigen selection is crucial for developing personalized vaccines. Delivery systems need refinement to balance stability, targeting, safety, and immune activation. In solid tumors, heterogeneity, immune editing, antigen loss, and a suppressive microenvironment limit responses. Future research should focus on improving mRNA design and delivery, as well as combining vaccines with ICIs, local therapies, cell therapies, and other immune strategies to turn non-T-cell-inflamed tumors into T-cell-inflamed ones and reduce immune escape. The future of mRNA cancer vaccines relies on integrated approaches that merge immunogenic antigens, adaptable delivery platforms, and TME modulation. Melanoma is especially promising for further study. As prediction algorithms, nanodelivery, and immunotherapy strategies advance, mRNA vaccines are expected to evolve toward more precise and scalable clinical use, becoming key components of personalized cancer immunotherapy.

## Glossary

ADCC: Antibody-Dependent Cellular Cytotoxicity
AEs: Adverse Events
APC: Antigen-Presenting Cell
ApoE: Apolipoprotein E
ARCA: Anti-Reverse Cap Analog
BBB: Blood-Brain Barrier
BCMA: B-Cell Maturation Antigen
CAR: Chimeric Antigen Receptor
**CD4** ^**+**^ **/CD8** ^**+**^: Cluster of Differentiation 4/8 Positive
cGAS: Cyclic GMP-AMP Synthase
circRNA: Circular RNA
CNS: Central Nervous System
COMPASS: Manganese-Coordinated Polyvalent Aptameric System
CRC: Colorectal Cancer
CRPC: Castration-Resistant Prostate Cancer
CRS: Cytokine Release Syndrome
CTL: Cytotoxic T Lymphocyte
CTLA-4: Cytotoxic T-Lymphocyte-Associated Protein 4
DC: Dendritic Cell
F-PEI: Fluoroalkyl-Grafted Polyethyleneimine
GBM: Glioblastoma Multiforme
GC: Gastric Cancer
GI: Gastrointestinal
GPC3: Glypican-3
HCC: Hepatocellular Carcinoma
HCMV: Human Cytomegalovirus
HER2: Human Epidermal Growth Factor Receptor 2
HLA: Human Leukocyte Antigen
HPV: Human Papillomavirus
HR+: Hormone Receptor-Positive
ICD: Immunogenic Cell Death
ICI/ICIs: Immune Checkpoint Inhibitor(s)
IDO1: Indoleamine 2,3-Dioxygenase 1
IFN: Interferon
IIMs: Innate Immune Modulators
IL: Interleukin
KRAS: Kirsten Rat Sarcoma Viral Oncogene Homolog
LAMP: Lysosome-Associated Membrane Protein
LNP: Lipid Nanoparticle
LPP: Lipopolyplex
MAGE-A3: Melanoma-Associated Antigen A3
MGMT: O6-Methylguanine-DNA Methyltransferase
MHC-I/MHC-II: Major Histocompatibility Complex Class I/II
MRD: Minimal Residual Disease
NK: Natural Killer (Cell)
NSCLC: Non-Small Cell Lung Cancer
NY-ESO-1: New York Esophageal Squamous Cell Carcinoma 1
OS: Overall Survival
OX40L: OX40 Ligand
PBAE: Poly(β-Amino Ester)
PD-1/PD-L1: Programmed Death-1/Programmed Death-Ligand 1
PDAC: Pancreatic Ductal Adenocarcinoma
PEG: Polyethylene Glycol
PKR: Protein Kinase R
PNPs: Polymeric Nanoparticles
POctS: Polyguanidinium Thioctic Acid
PSB: Photosynthetic Bacteria
RdRP: RNA-Dependent RNA Polymerase
RFS: Recurrence-Free Survival
RIG-I: Retinoic Acid-Inducible Gene I
saRNA: Self-Amplifying RNA
siRNA: Small Interfering RNA
SORT: Selective Organ Targeting
STING: Stimulator of Interferon Genes
TAA: Tumor-Associated Antigen
TAP: Transporter Associated with Antigen Processing
Tcm/Tem: Central/Effector Memory T Cell
Tfh: T Follicular Helper Cell
TIME: Tumor Immune Microenvironment
TLR: Toll-Like Receptor
TMB: Tumor Mutational Burden
TME: Tumor Microenvironment
TNBC: Triple-Negative Breast Cancer
TPTE: Transmembrane Phosphatase with Tensin Homology
TSA/Neoantigen: Tumor-Specific Antigen/Neoantigen
UTR: Untranslated Region
